# Engineering *Nannochloropsis oceanica* for the production of diterpenoid compounds

**DOI:** 10.1002/mlf2.12097

**Published:** 2023-12-26

**Authors:** Zhi‐Yan Du, Wajid W. Bhat, Eric Poliner, Sean Johnson, Conor Bertucci, Eva Farre, Bjoern Hamberger

**Affiliations:** ^1^ Department of Molecular Biosciences and Bioengineering University of Hawaii at Manoa Honolulu Hawaii USA; ^2^ Department of Biochemistry and Molecular Biology Michigan State University East Lansing Michigan USA; ^3^ Department of Plant Biology Michigan State University East Lansing Michigan USA; ^4^ Present address: New England Biolabs Inc. 240 County Road Ipswich 01938 MA USA

**Keywords:** casbene, microalgae, plastid targeting, synthetic biology, terpene synthase

## Abstract

Photosynthetic microalgae like *Nannochloropsis* hold enormous potential as sustainable, light‐driven biofactories for the production of high‐value natural products such as terpenoids. *Nannochloropsis oceanica* is distinguished as a particularly robust host with extensive genomic and transgenic resources available. Its capacity to grow in wastewater, brackish, and sea waters, coupled with advances in microalgal metabolic engineering, genome editing, and synthetic biology, provides an excellent opportunity. In the present work, we demonstrate how *N. oceanica* can be engineered to produce the diterpene casbene—an important intermediate in the biosynthesis of pharmacologically relevant macrocyclic diterpenoids. Casbene accumulated after stably expressing and targeting the casbene synthase from *Daphne genkwa* (DgTPS1) to the algal chloroplast. The engineered strains yielded production titers of up to 0.12 mg g^−1^ total dry cell weight (DCW) casbene. Heterologous overexpression and chloroplast targeting of two upstream rate‐limiting enzymes in the 2‐C‐methyl‐ d‐erythritol 4‐phosphate pathway, *Coleus forskohlii* 1‐deoxy‐ d‐xylulose‐5‐phosphate synthase and geranylgeranyl diphosphate synthase genes, further enhanced the yield of casbene to a titer up to 1.80 mg g^−1^ DCW. The results presented here form a basis for further development and production of complex plant diterpenoids in microalgae.

## Impact statement

Microalgae are tiny photosynthetic organisms that hold great potential in producing valuable natural compounds, such as terpenoids, with applications in pharmaceutical and other industries. In particular, engineered algae offer a unique advantage as a source of these compounds. Here, we demonstrate the production of casbene, a diterpene with antibacterial and antifungal properties, in the marine alga *Nannochloropsis* by multiplexing overexpression constructs. This breakthrough could open up new avenues for the production of important natural compounds using microalgae as a factory.

## INTRODUCTION

Terpenoids (also known as isoprenoids or terpenes) form the largest family of specialized metabolites, with over 84,000 known terpenoids reported across all kingdoms of life (Dictionary of Natural Products, DNP 30.2, March 1, 2022). These chemically, structurally, and functionally diverse classes of hydrocarbon molecules serve numerous biological roles, including defense, pigmentation, signaling, photoprotection, light harvesting, electron transfer, growth and development, and adaptation against abiotic and biotic stresses^1^, ^2^. In addition to their multitude of biological functions, terpenoids have a range of commercial applications, including pharmaceuticals, nutraceuticals, flavors/fragrances, food and feed additives, agricultural biocontrol agents, chemical feedstock, and biofuels^3^, ^4^, ^5^.

In photosynthetic organisms, terpenoids are derived from isopentenyl diphosphate (IPP) and dimethylallyl diphosphate (DMADP) precursors by the mevalonate (MVA) pathway, or the 2‐C‐methyl‐d‐erythritol‐4‐phosphate (MEP) pathway. The MVA pathway provides the precursors for the biosynthesis of sesquiterpenoids, triterpenoids, for example, phytosterols, brassinosteroids, and polyprenols. The MEP pathway is used for the biosynthesis of hemiterpenoids, monoterpenoids, diterpenoids, sesterterpenoids, cytokinins, gibberellins, carotenoids, tocopherols, and plastoquinones^6^. The MVA pathway operates in the cytosol of eukaryotes, while the MEP pathway is associated with bacteria and plant plastids^7^. In terpenoid biosynthesis, IPP and DMADP are sequentially condensed by prenyltransferases, which are geranyl diphosphate synthase, farnesyl diphosphate synthase, and geranylgeranyl diphosphate synthase yielding geranyl diphosphate (GDP, C10), farnesyl diphosphate (FDP, C15), and geranylgeranyl diphosphate (GDPP, C20), respectively^8^, ^9^, ^10^, ^11^, ^12^. These di‐phosphorylated carbon precursors are converted into a vast diversity of highly functionalized terpenoids by a range of enzymes, including terpene synthases, cytochromes P450, acyltransferases, and glycosyltransferases^13^, ^14^, ^15^, ^16^, ^17^, ^18^.

Specialized terpenoids often accumulate in small quantities or in mixtures of similar compounds (e.g., resins), which challenges extraction and purification from natural sources commercially, while chemical synthesis is limited due to the complexity of the structures^19^, ^20^, ^21^. Also, some of the high‐value terpenoids are found in potentially rare, endemic, or endangered organisms, and their overexploitation can be detrimental to those species^22^, ^23^. The modular nature of terpenoid metabolism and ubiquitous production of IPP and DMADP in all living organisms, combined with the high demand for terpenoids, makes their heterologous production particularly appropriate to metabolic engineering approaches^13^, ^24^, ^25^, ^26^. There have been many successful examples of metabolic engineering that have given access to a range of biotechnologically relevant terpenoids and enhanced our understanding of recombinant pathways in heterologous hosts^27^, ^28^, ^29^, ^30^, ^31^.

Bacteria and yeasts have been the most popular heterologous hosts for terpenoid production as these organisms are easily amenable to genetic engineering and can be scaled up to industrial levels^32^, ^33^. However, these heterotrophic microbial systems rely on feeding with an external carbon source such as glucose, acetate, methanol, or pyruvate^34^, ^35^, ^36^. Recently, there has been a growing interest in the development of sustainable, light‐driven photosynthetic organisms as alternative terpenoid platforms^37^, ^38^, ^39^, ^40^, ^41^, ^42^, ^43^, ^44^. Photosynthetic microbes such as *Cyanobacteria* and eukaryotic microalgae like *Chlamydomonas reinhardtii* and *Nannochloropsis* spp. only require sunlight and carbon dioxide and thus offer the potential for sustainable production processes^38^, ^45^, ^46^.

*Nannochloropsis* is a genus of fast‐growing marine microalgae that are particularly amenable to metabolic and regulatory engineering^47^, ^48^, ^49^, ^50^, ^51^, ^52^, ^53^, ^54^. This genus has emerged as an exciting industrial model owing to its ability to grow rapidly under varied conditions, for example, in open ponds and photobioreactors using seawater, and was shown to synthesize and sequester large amounts of triacylglycerols and high‐value polyunsaturated fatty acids (up to 60% of their dry weight) in specialized compartments, lipid droplets^55^. These lipid reserves can be converted into biodiesel by trans‐esterification^56^, or may be targeted to efficiently sequester terpene products and anchor their biosynthetic routes^57^. *N. oceanica* is distinguished as a particularly robust transgenic host with extensive genomic resources and genetic tools available^47^, ^53^, ^54^, ^58^, ^59^, ^60^. Although *Nannochloropsis* spp. has been recently engineered for producing designer oils^48^, ^49^, there are no reports regarding the application of high‐value terpenoids.

**Figure 1:**
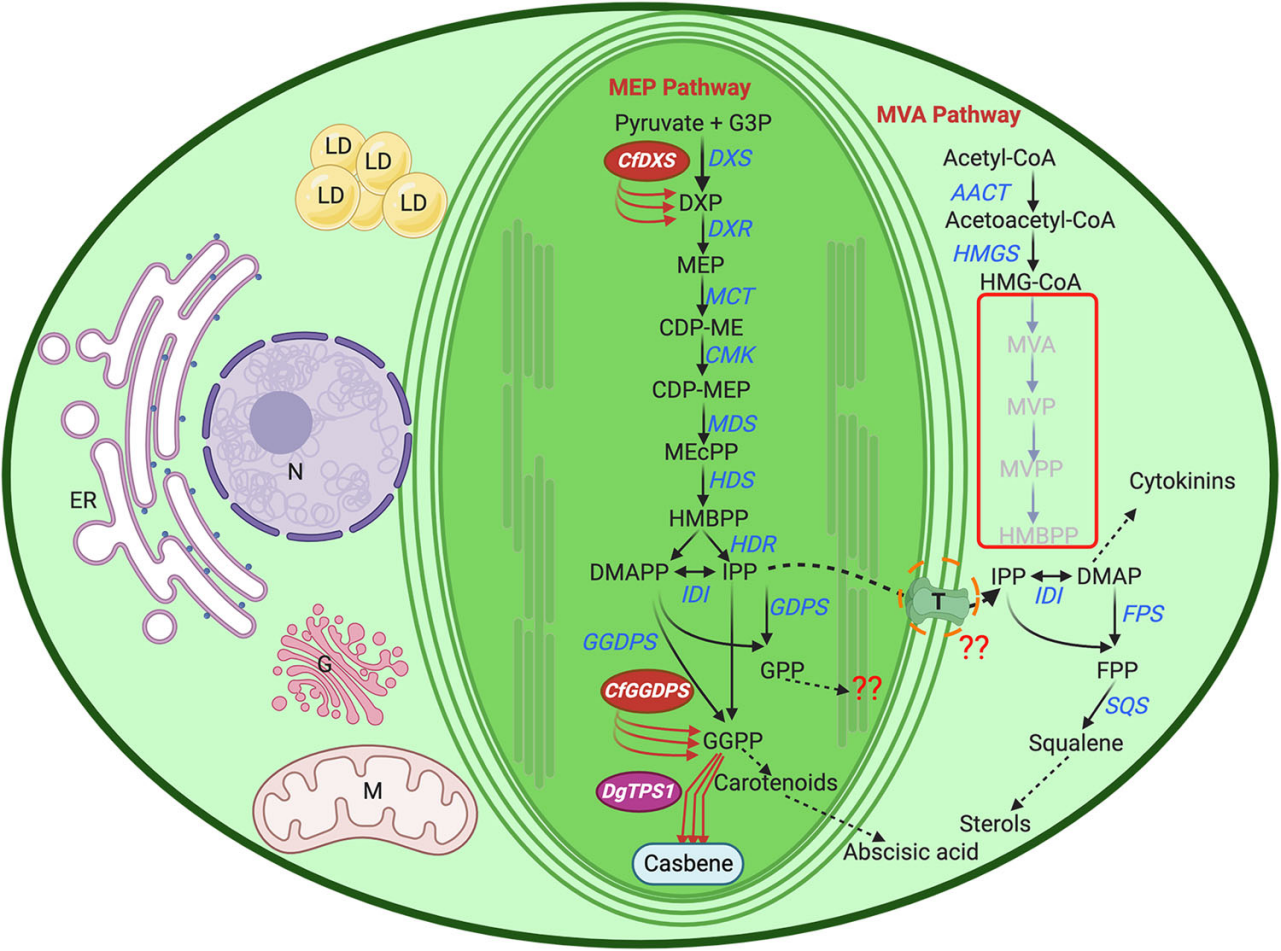
Biosynthesis pathways of terpenoids in *Nannochloropsis oceanica*. The MVA pathway is lacking in *N. oceanica*, including four key genes encoding hydroxy‐3‐methylglutaryl coenzyme A reductase (HMGR), mevalonate kinase (MVK), phosphomevalonate kinase (PMK), and mevalonate diphosphate decarboxylase (PMD). The missing steps are indicated by light gray color, and the products are highlighted by red box. AACT, acetoacetyl‐CoA thiolase; DMAP, 4‐dimethylaminopyridine; FPP, farnesyl diphosphate; FPS, farnesyl diphosphate synthase; HMBPP, 4‐hydroxy‐3‐methylbut‐2‐enyl diphosphate; HMG‐CoA, 3‐hydroxy‐3‐methylglutaryl‐CoA; HMGS, HMG‐CoA synthase; IDI, isopentenyl diphosphate isomerase; IPP, isopentenyl diphosphate; MVA, mevalonate; MVP, 5‐phosphomevalonate; MVPP, 5‐diphosphomevalonate; SQS, squalene synthase. The MEP pathway forms the terpenoid precursors in the chloroplast of *N. oceanica*. CDP‐ME, 4‐(cytidine 5′‐diphospho)‐2‐C‐methyl‐d‐erythritol; CDP‐MEP, 4‐(cytidine 5′‐diphospho)‐2‐C‐methyl‐d‐erythritol phosphate; DXP, 1‐deoxy‐d‐xylose 5‐phosphate; DXS, DXP synthase; DXR, MEP synthase; ER, endoplasmic reticulum; G, Golgi apparatus; G3P, glyceraldehyde 3‐phosphate; GGDPS/GGPPS, geranylgeranyl diphosphate synthase; GGPP, geranylgeranyl diphosphate; GPP, geranyl diphosphate; HDR, 4‐hydroxy‐3‐methylbut‐2‐enyl diphosphate reductase; HDS, 4‐hydroxy‐3‐methylbut‐2‐enyl diphosphate synthase; LD, lipid droplet; M, mitochondria; MCT, 2‐C‐methyl‐d‐erythritol 4‐phosphate cytidylyltransferase; MEP, 2‐C‐methyl‐D‐erythritol 4‐phosphate; MDS, 2‐C‐methyl‐d‐erythritol 2,4‐cyclodiphosphate synthase; MEcPP, 2‐C‐methyl‐d‐erythritol 2,4‐cyclodiphosphate; N, nucleus; TPS, terpene synthase. Red arrows indicate heterologously expressed *CfDXS*, *CfGGDPS* (*Coleus forskohlii*), and *DgTPS1* (*Daphne genkwa*).

**Figure 2:**
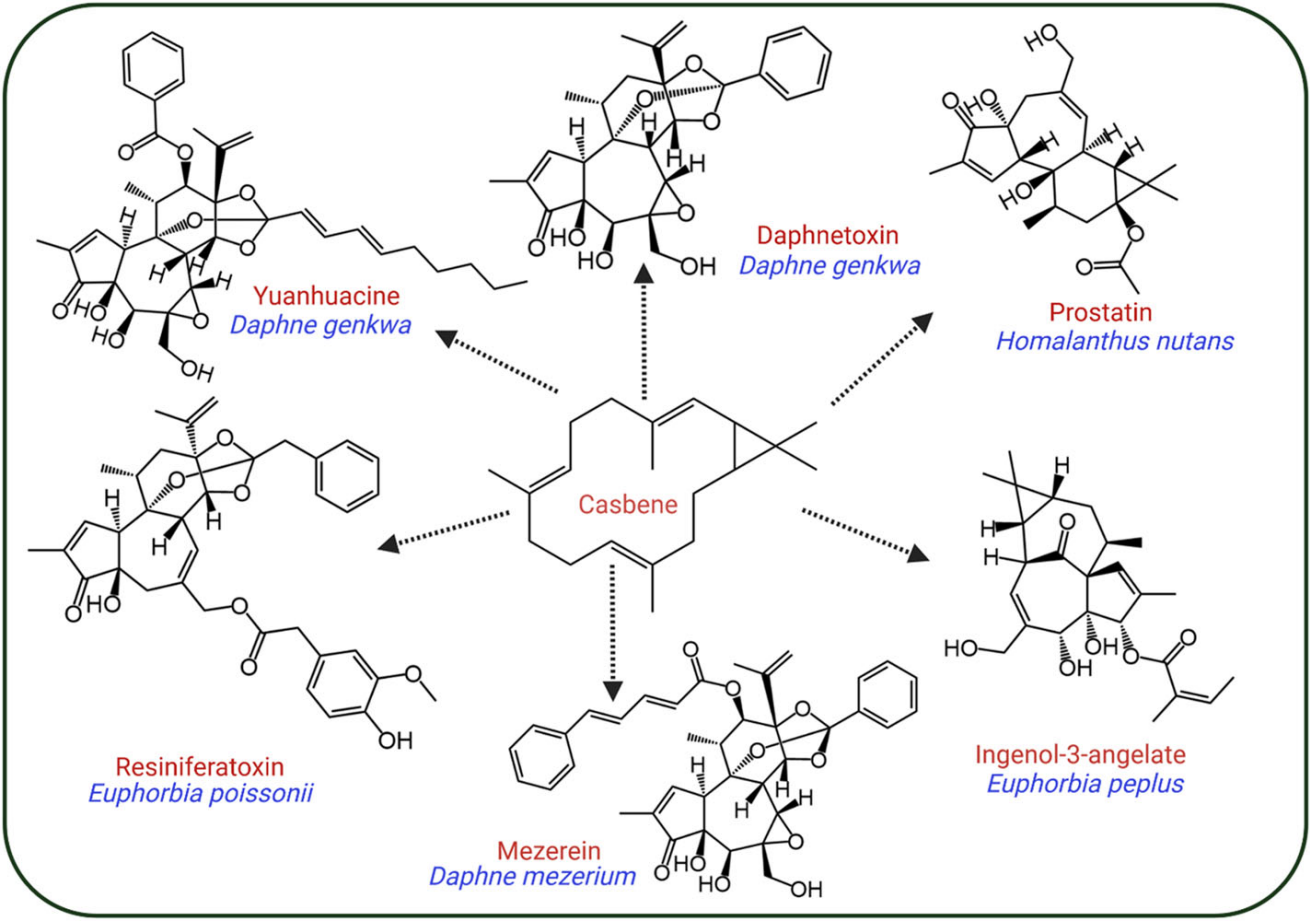
Using casbene as a model diterpene for the synthesis of high‐value terpenes. Key and representative specific products (in red) are shown in plant species (in blue). Casbene is important for the biosynthesis of other diterpenes, such as Yuanhuacine and anticancer Daphnetoxin in *Daphne genkwa*, anti‐HIV prostatin in *Homolanthus nutans*, antitumor Ingenol‐3‐angelate in *Euphorbia peplus*, toxic Mezerein in *Daphne mezerium*, and vanilloid receptor agonist Resiniferatoxin in *Euphorbia poissonii*.

*N. oceanica*, like many microalgal species, lacks a functional MVA pathway including four key genes encoding hydroxy‐3‐methylglutaryl coenzyme A reductase (HMGR), mevalonate kinase (MVK), phosphomevalonate kinase (PMK), and mevalonate diphosphate decarboxylase (PMD) (Figure 1)^61^. Therefore, the native MEP pathway supplies all the cellular precursors needed for the biosynthesis of pigments associated with their color and photosynthetic machinery^11^. The terpenoid precursors found in *N. oceanica* are produced exclusively in the chloroplast and are transported to the cytosol and other organelles via yet unknown transporter proteins, where they are utilized for the biosynthesis of cholesterol and other related phytosterols (Figure 1). Through photosynthesis, microalgae like *N. oceanica* naturally generate a significant pool of isoprenoid products, including the C20 precursor of carotenoids and the phytol chain of chlorophyll molecules, geranylgeranyl diphosphate (GGPP), and thus present an exciting opportunity for metabolic engineering of terpenoids, in particular, diterpenoids. In this work, we sought to hijack the GGPP‐producing machinery of *N. oceanica* for the production of casbene, a central intermediate in the biosynthesis of high‐value diterpenoids^17^. These include the pharmacologically active vanilloid receptor (VR) agonist Resiniferatoxin from *Euphorbia poissonii*, the anticancer Daphnetoxin and Yuanhuacine from *Daphne genkwa*, the toxic Mezerein in *Daphne mezerium*, the anti‐HIV prostatin in *Homolanthus nutans*, and the antitumor Ingenol‐3‐angelate in *Euphorbia* ssp. (Figure 2). The goal of this study was to determine the potential of *N. oceanica* as a stable system for diterpenoid production and to lead the way forward for multigene and multicompartment engineering of *N. oceanica*. We demonstrate robust production of casbene by targeting *D. genkwa* casbene synthase (DgTPS1) into the chloroplasts of *N. oceanica* using the native violaxanthin/chlorophyll *a*‐binding protein 1 (NoVCP1) N*‐*terminal plastid targeting sequence. The capacity of *N. oceanica* to produce casbene was further boosted by overexpression of plastid‐targeted 1‐deoxy‐d‐xylulose‐5‐phosphate synthase (CfDXS) and geranylgeranyl diphosphate synthase (CfGGPPS) sourced from *Plectranthus barbatus* (syn. *Coleus forskohlii*).

## RESULTS AND DISCUSSION

### Selection of subcellular compartment for terpene production

**Figure 3:**
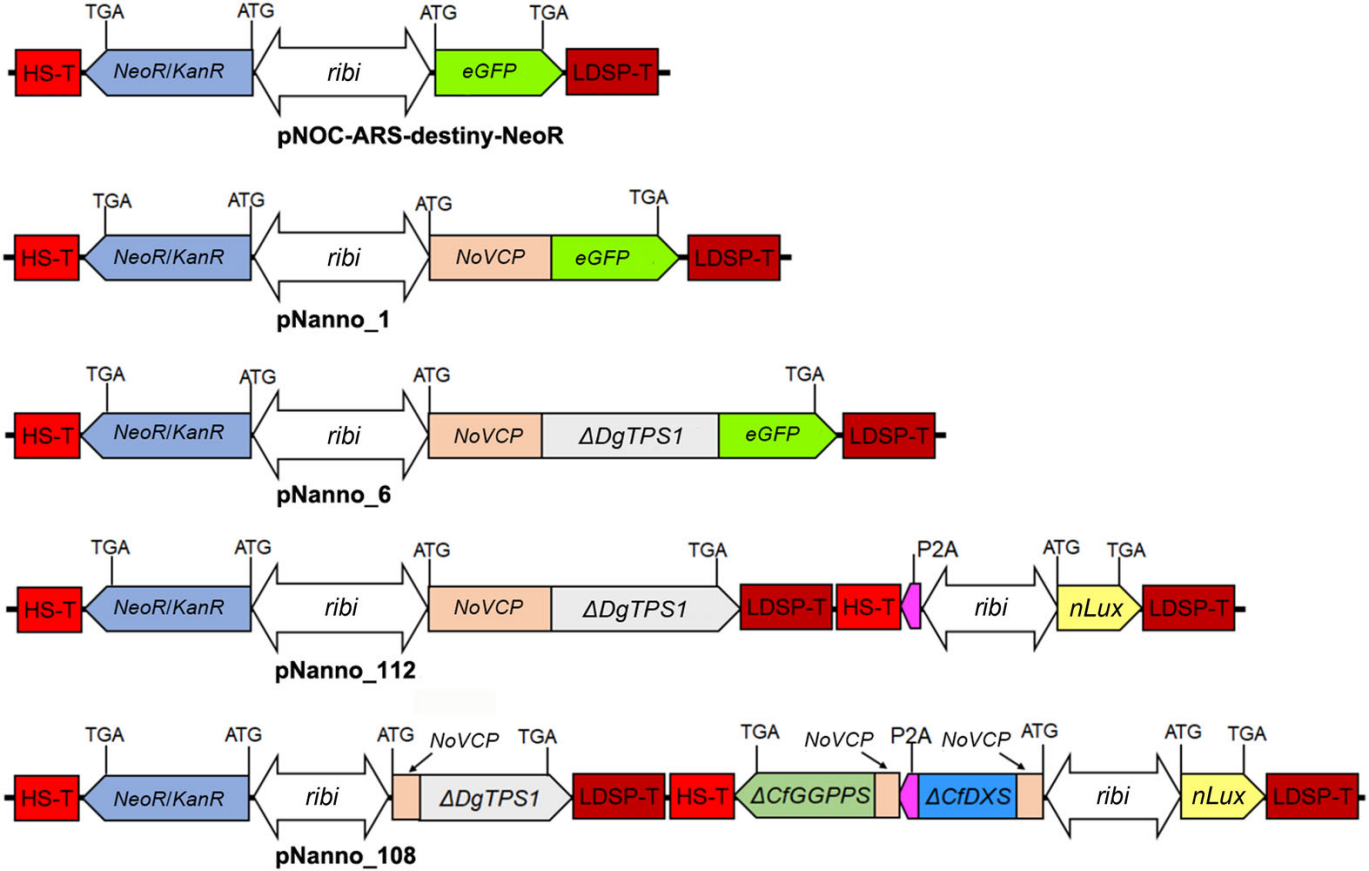
Overview of the constructs in this study. *eGFP*, green fluorescent protein gene; LDSP‐T, lipid droplet surface protein gene terminator; *NeoR/KanR*, G418 resistance gene; *nLux*, nanoLuciferase gene; *NoVCP*, N‐terminal plastid targeting sequence of *Nannochloropsis oceanica* violaxanthin/chlorophyll *a*‐binding protein 1 gene; *ribi*, ribosomal subunit bidirectional promoter; *ΔDgTPS1*, functionally verified pseudomature casbene synthase gene; *Δ25_DgTPS1*; *ΔCfDXS*, *Coleus forskohlii* 1‐deoxy‐d‐xylulose‐5‐phosphate synthase gene *Δ40_CfDXS*; *ΔCfGGPPS*, geranylgeranyl diphosphate synthase gene *Δ43_CfGGPPS*.

**Figure 4:**
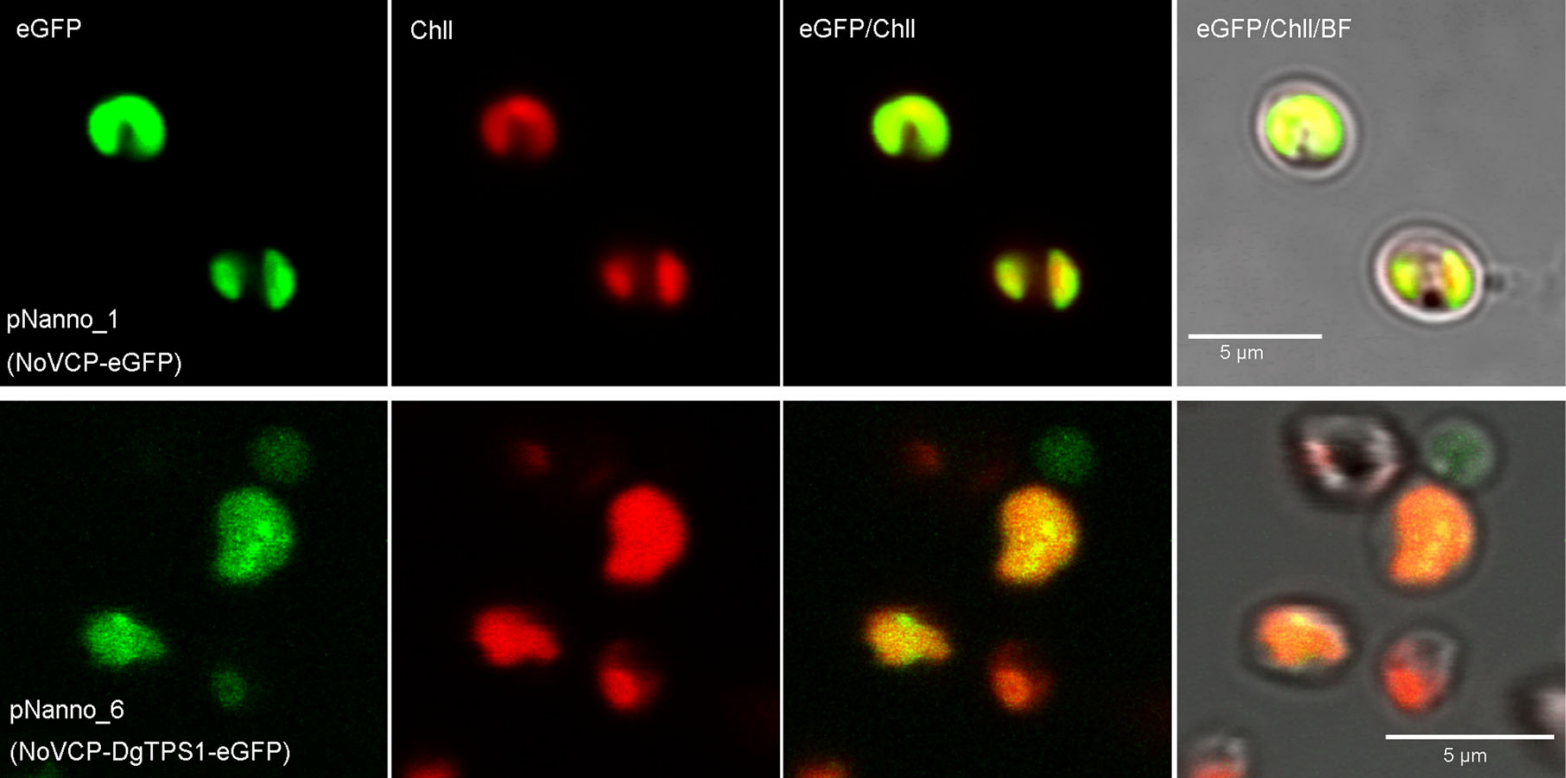
Localization of the *Daphne genkwa* casbene synthase (DgTPS1) in the chloroplast by confocal microscopy. NoVCP, N‐terminal plastid targeting sequence from the *Nannochloropsis oceanica* violaxanthin/chlorophyll *a*‐binding protein 1. Details of the two constructs pNanno_1 and pNanno_6 are listed in Figure 3. BF, bright field; Chll, autofluorescence of chloroplast in red.

*N. oceanica* being a heterokont, has a complex chloroplast compared to that of chlorophyte algae and higher plants because it is surrounded by four membranes derived from the secondary endosymbiosis of a red alga^62^. The outermost plastid membrane is connected to the outer nuclear envelope membrane to form a nucleus‐plastid continuum^63^, as illustrated in Figure 1. We thus reasoned that the plant plastid targeting sequence that works efficiently with higher plant plastids was nonfunctional in *N. oceanica*. We selected the NoVCP1 to construct recombinant fusion proteins for importing into the plastid (Figure S1). NoVCP1 is a component of the light‐harvesting complexes at the thylakoid membrane and resides inside the chloroplasts of *Nannochloropsis* ^64^. The subcellular targeting prediction method HECTOR^65^ predicted the first 33 amino acids in NOVCP1 to be the plastid targeting sequence. To functionally verify this, we fused the 33 N‐terminal amino acids of NoVCP1 to eGFP and transformed *N. oceanica* (Figure 3). Fluorescent microscopy confirmed that eGFP was targeted exclusively into the chloroplast of *N. oceanica* (Figure 4, pNanno_1). As control and for compartment‐specific targeting, we also targeted eGFP to the *N. oceanica* endoplasmic reticulum (ER) and mitochondria by fusing it with the signal/transit peptides of the mitochondrial oxidase assembly protein1 (NoOXA1) and ER protein disulfide isomerase (NoPDI), as shown in Figure S1. It has been previously shown that some terpene synthases can be fused to fluorescent reporters without affecting the catalytic properties of the enzyme^38^, ^66^. To confirm the subcellular localization of Δ25DgTPS1, the *NoVCP1‐TP*_*ΔDgTPS1* gene was expressed in frame with a C‐terminal eGFP (pNanno_6). The resulting heterologous fusion protein was detected in *N. oceanica* chloroplasts by fluorescence microscopy (Figure 4).

### Heterologous expression of *D. genkwa* casbene synthase (DgTPS1) produces casbene in *N. oceanica* chloroplast

**Figure 5:**
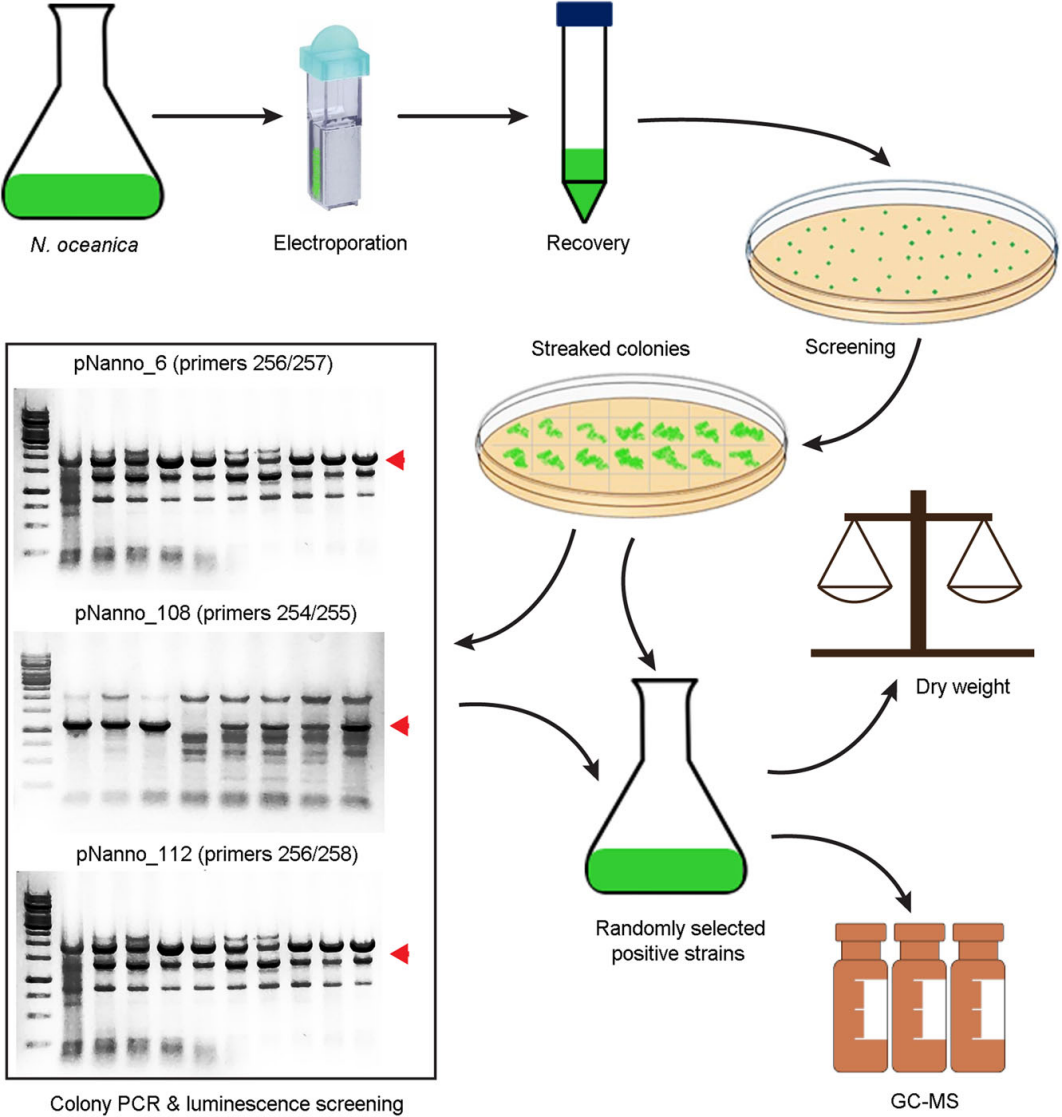
Overview of the transformation and mutant screening. Details are explained in the Materials and Methods section. The red arrowhead highlights bands characteristic for positive genotyping by PCR. The sequences of primers for colony PCR are listed in Table S1. Luminescence screening results are shown in Figure S3. GC‐MS, gas chromatography‐mass spectrometry.

**Figure 6:**
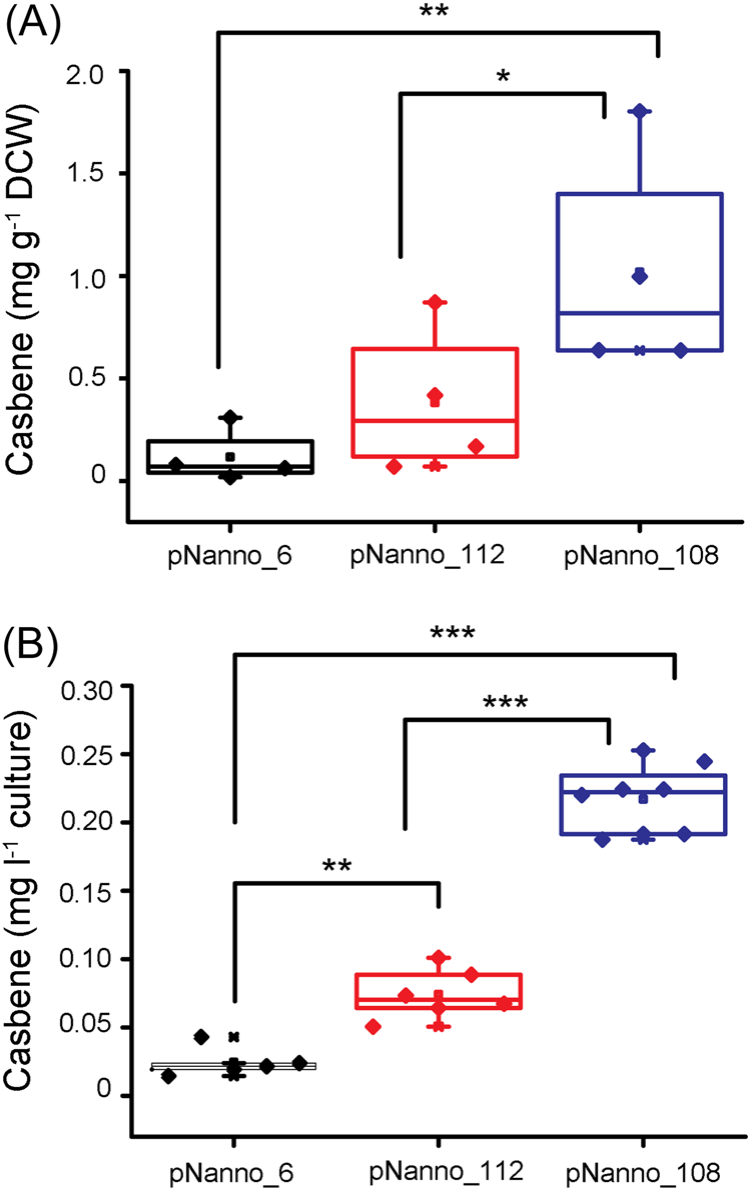
Casbene production in the *Nannochloropsis oceanica* transformants. Construct details are shown in Figure 3. Using GC‐MS, the production of casbene by dry cell weight (DCW, A) and culture volume (B) was determined in pNanno_6 (*Δ25DgTPS1*‐C‐terminal *eGFP*), pNanno_112 (*Δ25DgTPS1*), and pNanno_108 (*Δ25DgTPS1*, *Δ40CfDXS*, and *Δ43CfGGPPS*). Solid diamonds are the average of casbene production of three to six independent transformants of each construct, with three biological replicates for each transformant. Horizontal lines within the box represent the median values. Asterisks indicate significant differences between the samples based on the *t*‐test. *p ≤ 0.05; **p ≤ 0.01; ***p ≤ 0.001.

After confirming the chloroplastic localization of NoVCP1 targeting peptide using NoVCP1 TP‐eGFP fusion protein, we tested the *N. oceanica* system for the production of diterpenes following the steps in Figure 5. As a proof of concept, we selected a class I diterpene synthase from *D. genkwa*, casbene synthase (DgTPS1; accession number: MZ485349.1). Casbene synthases catalyze the formation of the macrocyclic diterpene casbene, a central intermediate in the biosynthesis of lathyrane, tigliane, ingenane, and daphnane diterpenes^17^. We fused the Δ25_DgTPS1, lacking its native plant‐specific plastidic localization signal peptide, with the functionally validated heterokont‐specific NoVCP1 signal/transit peptide (pNanno_112). The resulting construct *NoVCP1*_*ΔDgTPS1* was transformed into *N. oceanica* cells. Positive transformants were confirmed by colony PCR (Figure S2), screened with Luminescence assays (Figure S3), and confirmed the expression of *CfDXS* and *CfGGPPS* in pNanno_108 (Figure S4). Gas chromatography‐mass spectrometry (GC‐MS) confirmed the production of casbene in the transgenic lines (Figure 6), while no casbene was found in *N. oceanica* lines transformed with an empty vector (Figure S5).

### Co‐expression of *P. barbatus* DXS (CfDXS) and (CfGGPPS) increases casbene production in *N. oceanica* chloroplast

To further improve the production of casbene in *N. oceanica*, we performed multigene engineering by adding the upstream pathway genes, *P. barbatus* (formerly known as *C. forskohlii)* deoxyxylulose 5‐phosphate synthase gene (*Δ40_CfDXS*), and geranylgeranyl diphosphate synthase gene (*Δ43_CfGGPPS*), encoding rate‐limiting enzymes of the diterpenoid biosynthesis^11^ targeted to the chloroplast (Figure 3). DXS is the first enzyme in the MEP pathway, whereas GGPPS catalyzes the formation of GGPP from IPP and DMADP. Co‐expression of *CfDXS* and *CfGGPPS* have been shown to substantially increase the level of the substrate GGPP^15^, ^39^, ^67^, ^68^ (Figure 1). A series of strains overexpressing and targeting *DgTPS1*, *CfDXS*, and *CfGGPPS* to the *N. oceanica* chloroplast was generated: pNanno_108 (*Δ25_DgTPS1*, *Δ40_CfDXS*, and *Δ43_CfGGPPS*), pNanno_112 (*Δ25_DgTPS1*), and pNanno_6 (*Δ25_DgTPS1:eGFP*) (Figure 2). Successful transformants were obtained for all genes, as confirmed by genotyping (Figure S2). Expression of *CfDXS* and *CfGGPPS* in pNanno_108 transformants was confirmed with qRT‐PCR (Figure S4), which further increased the production of casbene up to 1.80 mg g^−1^ DCW (Figure 6A) and 0.25 mg l^−1^ culture (Figure 6B). The significant increase upon *CfDXS* and *CfGGPPS* overexpression is in the same range as previously reported for heterologous production of other terpenoids such as carotenes and monoterpenes in *Synechocystis* sp. PCC 6803 with overexpression of the endogenous *DXS* and *GGPPS* ^67^, ^69^, ^70^, indicating that either the native *N. oceanica* DXS and GGPPS may be subjected to regulation, which may not apply to the enzymes sourced from the plants, or multiple copies of *DXS* and *GGPPS* may have an additive effect on the GGPP accumulation. Nevertheless, casbene titers reached in the highest‐producing strain (pNanno_108_8) are comparable to those achieved for other terpenoids in *Cyanobacteria*, such as 0.25 mg g^−1^ DCW of isoprene, 0.45 mg g^−1^ DCW of 13R‐manoyl oxide, and 0.36 mg g^−1^ DCW of geranyllinalool in *Synechocystis* ^67^, ^71^, ^72^.

There are multiple opportunities for improving the terpenoid yields in *N. oceanica*. The genes used for expression in this study were not codon‐optimized for *N. oceanica*, which has previously been shown to enhance the expression of a plant enzyme in *Synechocystis* and *C. reinhardtii* ^38^, ^39^, ^73^. Using the gene‐stacking constructs, more candidate genes such as the *Arabidopsis* 4‐hydroxy‐3‐methylbut‐2‐enyl diphosphate reductase gene *AtHDR* and *Jatropha curcas* casbene synthase gene *JcCAS* could be used to increase productivity^74^. To reach maximal productivities, the growth conditions could be further optimized: the growth period could be extended to achieve higher cell densities/biomass using photobioreactors^55^; stress conditions such as nitrogen starvation could be used to stimulate the lipid biosynthesis^49^, ^55^; solvents such as dodecane could be used to simulate and collect the casbene excreted from the algae^39^, ^75^. In addition, stronger promoters may be tested to increase the yield. We plan to perform extensive multi‐gene and multicompartment engineering (e.g., promoters, transcription factors, and enzymes) in the future to tap into the lipid droplets as organelles for diterpenoid production and sequestration.

In conclusion, the ability to produce nonnative diterpenoids in *N. oceanica* opens the possibility for the development of sustainable light‐driven biofactories for a vast range of chemicals from *Nannochloropsis* sp. We demonstrate here that the chloroplast localized MEP pathway in *N. oceanica* can be tapped to produce nonnative diterpenoid molecules and that channeling carbon toward the desired product does not affect the fitness of *N. oceanica*. In addition, the demonstration that overexpressing the upstream pathway genes improves the production of casbene in *N. oceanica* here is the first step toward multigene engineering for terpenoid compounds from this host. Similar strategies built on the results presented in this work can be applied for future synthetic biology‐guided engineering processes and other microalgal hosts for light‐driven production of a range of high‐value diterpenoids.

## MATERIALS AND METHODS

### Strains, media, and maintenance

*N. oceanica* CCMP1779, the marine alga used in this study, was obtained from the Provasoli‐Guillard National Center for Culture of Marine Phytoplankton. The algal cells were incubated as described previously^76^. In brief, f/2 medium (2.5 mM NaNO_3_, 0.036 mM NaH_2_PO_4_, 0.106 mM Na_2_SiO_3_, 0.012 mM FeCl_3_, 0.012 mM Na_2_EDTA, 0.039 mM CuSO_4_, 0.026 mM Na_2_MoO_4_, 0.077 mM ZnSO_4_, 0.042 mM CoCl_2_, 0.91 mM MnCl_2_, 0.3 mM thiamine HCl/vitamin B1, 2.05 nM biotin, 0.37 nM cyanocobalamin/vitamin B_12_, 15 mM Tris buffer, pH 7.6) was used to grow the alga with 20 mM sodium bicarbonate as the extra carbon supplement. The cells were grown in batch cultures in flasks or 96 deep well plates (Evergreen) on a shaker (120 rpm) at 23°C, under continuous light at ~100 mmole photons m^−2^ s^−1^ (CRI 98 LED lights by YUJILEDS, Cat No. YJ‐VTC‐RB‐2835‐12‐56). Cell size and density of cultures were calculated using a Z2 Coulter Counter (Beckman).

### Plasmid construction, transformation, and screening of mutants

Cloning in this work was conducted using NEB restriction enzymes and an Infusion cloning kit (Clontech Labs 3P‐639648) according to the manufacturer's protocols. Phusion high‐fidelity polymerase (F‐530; Thermo Scientific) was used to perform PCRs following the manufacturer's protocols. The primers used are listed in Table S1. Plasmid vectors pNOC‐ARS‐stacked‐NeoR‐GFP, pNOC‐stacked‐MCS‐Nlux, and pNOC‐ARS‐destiny‐NeoR^59^ were used for creating the constructs. Their maps and sequences are provided as Supporting Information. For localization studies, the sequences coding for signal/transit peptides of NoVCP1, NoPDI, and NoOxa1 were amplified from the *N. oceanica* genomic DNA and cloned into pNOC‐ARS‐stacked‐NeoR‐GFP as N‐terminal GFP fusion proteins for plastid (pNanno_1), endoplasmic reticulum (pNanno_2) and mitochondrial (pNanno_3) localization, respectively. A functionally verified pseudomature casbene synthase gene (*Δ25_DgTPS1*; accession number: MZ485349.1) lacking the plant‐specific transit peptide, was amplified from *D. genkwa* cDNA and cloned into pNanno_1 between the NoVCP1 signal/transit peptide and eGFP, to create pNanno_6. For plastid targeting of casbene synthase, the NoVCP1 signal/transit peptide and *Δ25_DgTPS1* were amplified and cloned into pNOC‐ARS‐destiny‐NeoR plasmid (pNanno_101). LR recombination reaction (Gateway LR Clonase II Enzyme mix, 11791100; Invitrogen) between pNanno_101 and pNOC‐stacked‐MCS‐Nlux created the final construct pNanno_112. For multigene engineering, 1‐deoxy‐d‐xylulose‐5‐phosphate synthase gene (*Δ40_CfDXS*; NCBI ID KP889115.1) and geranylgeranyl diphosphate synthase gene (*Δ43_CfGGPPS*; NCBI ID KP889114) lacking the plant‐specific transit peptide were amplified and sequentially cloned into pNOC‐stacked‐MCS‐Nlux with the N‐terminal NoVCP1 signal/transit peptide to create pNanno_106. Δ40_CfDXS and Δ43_CfGGPPS were separated by a P2A linker protein that has been shown to work efficiently in *N. oceanica* ^59^. The final construct pNanno_108 was created by an LR recombination reaction between pNanno_101 and pNanno_106 as per the Gateway cloning protocol. Plant and heterokont‐specific signal/transit peptides were predicted by TargetP https://services.healthtech.dtu.dk/services/TargetP-2.0/) and HECTAR (https://webtools.sb-roscoff.fr/) programs, respectively. All vector sequences were confirmed by sequencing (Psomagen NGS). Plasmids were maintained in *Escherichia coli* DH5α or ccdB‐resistant DB3.1. The plasmid maps are provided as Supporting Information. Oligonucleotides used for cloning are listed in Table S1.

Algae transformation was performed according to the published protocol^55^. The log‐phase algal cells were washed three times with 375 mM sorbitol (56755; Sigma‐Aldrich) and then resuspended in the fresh sorbitol buffer at 500 million cells ml^−1^. Two hundred microliters of the sample was used for transformation by electroporation. Two to three micrograms of linearized plasmids (without blocking DNA) were added to the samples, followed by electroporation in a 2 mm cuvette (BioRad) in a Gene Pulser II (BioRad), with an exponential decay pulse (2.2 kV, 600 Ω, 50 μF). Transformants were recovered for 48 h in 5 ml of f/2 medium in 15 ml conical tubes with shaking under constant light (100 rpm, ~100 μmol m^−2^ s^−1^) at room temperature. Cell cultures were then mixed with 5 ml of top agar (f/2 medium, 0.5% agar, 35°C) and spread onto f/2 selection plates containing G418 (250 mg l^−1^, VWR), and colonies were picked after 1 month of cultivation.

### Colony PCR

Cells of transformants were resuspended in 20 μl of 1× PCR buffer (Phire Plant Direct PCR Kit, F130WH; Thermo Scientific). The cell resuspension was cultivated at 98°C for 10 min, and then centrifuged for 1 min (10,000*g*). Subsequently, 2 μl of supernatant was used as the DNA template for 20 μl PCR reactions.

### Confocal microscopy

Confocal microscopy was conducted to investigate the localization of DgTPS1 (pNanno_106, DgTPS1::eGFP). The samples were observed using an Olympus Spectral FV1000 microscope with an argon (488 nm) laser for eGFP (emission, 510–530 nm) and a solid‐state laser (556 nm) for chloroplast (emission, 655–755 nm).

### Luminescence screening assays

Luminescence screening was performed following the previously published protocol^59^ with modifications. In brief, the cells of transformants and controls were incubated in 96 deep well plates, and 100 μl of the cell culture was transferred to the 96‐well reading plate and mixed with 100 μl of f/2 medium containing firefly luciferin (final concentration 500 μM; GoldBio) and NanoGlo substrate (N1110, 20,000 dilutions; Promega). Luminescence assays were conducted using a spectrofluorometer (SpectraMax M2). The luminescence readings were normalized by the cell density measurements at OD_750_.

### RNA assay

RNA was isolated with the RNeasy plant mini kit (QIAGEN) for reverse transcription (Superscript II reverse transcriptase; Invitrogen) to obtain cDNA for quantitative real‐time PCR (qRT‐PCR), using SYBR Green Master Mix (Life Technologies) and a StepOne Real‐Time PCR System (Applied Biosystems). Relative gene expression was obtained by the $2^{-∆∆C_{t}}$ method with *Nannochloropsis ACTIN* as the reference gene. Primers are listed in Table S1.

### GC‐MS analysis

Independent positive transformants of each construct were selected by the colony PCR and luminescence screening assays and then cultured for GC‐MS analysis with three biological replicates. The algal cells were harvested with liquid nitrogen and homogenized (Tissue Lyser II; QIAGEN). Total lipids were extracted with methanol‐chloroform‐formic acid (1:2:0.1, v/v/v). The samples were dried with nitrogen gas and resuspended in hexane for GC‐MS analyses, using an Agilent 7890A GC with an Agilent VF‐5ms column (30 m × 250 µm × 0.25 µm, with 10 m EZ‐Guard) and an Agilent 5975C mass selective detector. The inlet was set to 275°C splitless injections of 1 µl, He carrier gas with a column flow of 1 ml min^−1^. The detector was activated after a 4‐min solvent delay. The oven temperature was programmed to either be held at 80°C for 0.5 min, to increase 50°C min^−1^ to 250°C, followed by increases of 10°C min^−1^ to 280°C, 50°C min^−1^ to 320°C with a final hold for 4 min or an initial hold at 40°C for 1 min, followed by sequential increases of 40°C min^−1^ to 200°C, held for 5 min, 10°C min^−1^ to 280°C, and a final ramp of 40°C min^−1^ to 320°C, held by 3 min.

## AUTHOR CONTRIBUTIONS

**Zhi‐Yan Du**: Conceptualization (lead); data curation (lead); formal analysis (lead); funding acquisition (lead); investigation (lead); methodology (equal); project administration (lead); resources (lead); software (lead); supervision (lead); validation (lead); visualization (lead); writing—original draft (equal); writing—review and editing (lead). **Wajid W. Bhat**: Conceptualization (lead); data curation (lead); formal analysis (lead); funding acquisition (equal); investigation (equal); methodology (equal); project administration (equal); resources (lead); software (lead); supervision (equal); validation (lead); visualization (lead); writing—original draft (lead); writing—review and editing (lead). **Eric Poliner**: Conceptualization (supporting); investigation (supporting); methodology (supporting); resources (supporting); software (supporting); writing—review and editing (supporting). **Sean Johnson**: Conceptualization (supporting); investigation (equal); methodology (supporting); resources (supporting); software (supporting); writing—review and editing (supporting). **Conor Bertucci**: Data curation (supporting); investigation (supporting); methodology (supporting); writing—original draft (supporting); writing—review and editing (supporting). **Eva Farre**: Conceptualization (supporting); formal analysis (supporting); investigation (supporting); methodology (supporting); project administration (supporting); resources (supporting); validation (supporting); writing—original draft (supporting); writing—review and editing (supporting). **Bjoern Hamberger**: Conceptualization (equal); data curation (equal); formal analysis (equal); funding acquisition (equal); investigation (equal); methodology (equal); project administration (equal); resources (equal); software (equal); supervision (equal); validation (equal); visualization (equal); writing—original draft (equal); writing—review and editing (equal).

## ETHICS STATEMENT

This article does not contain any studies with human participants or animals performed by any of the authors.

## CONFLICT OF INTERESTS

The authors declare no conflict of interests.

## Supporting information

Supporting information.

Supporting information.

## Data Availability

The data that support the findings of this study are available in the Supporting Information material of this article.
