# Supplementary material for: Engineering Nannochloropsis oceanica for the production of diterpenoid compounds
Source: mLife. 2023 Dec 26;2(4):428–37. doi: 10.1002/mlf2.12097 (PMC10989085; doi:10.1002/mlf2.12097)
Supplement: Supplementary file 1 — Supporting information. [file MLF2-2-428-s001.pdf]

Engineering *Nannochloropsis oceanica* for the production of diterpenoid compounds

Zhi-Yan Du[1*ϕ], Wajid Waheed Bhat[2*], Eric Poliner[3], Sean Johnson[2#], Conor Bertucci[2], Eva Farre[3] and Bjoern Hamberger[2ϕ*]

[1]Department of Molecular Biosciences and Bioengineering, University of Hawaii at Manoa, [2]Department of Biochemistry and Molecular Biology, [3]Department of Plant Biology, Michigan State University.

[#]Present address: New England Biolabs, Inc., 240 County Road, Ipswich, MA 01938, USA.

[*]Equal contribution

[ϕ]For Correspondence: Bjoern Hamberger (hamberge@msu.edu) and Zhi-Yan Du (duz@hawaii.edu)

1. Supplemental figures

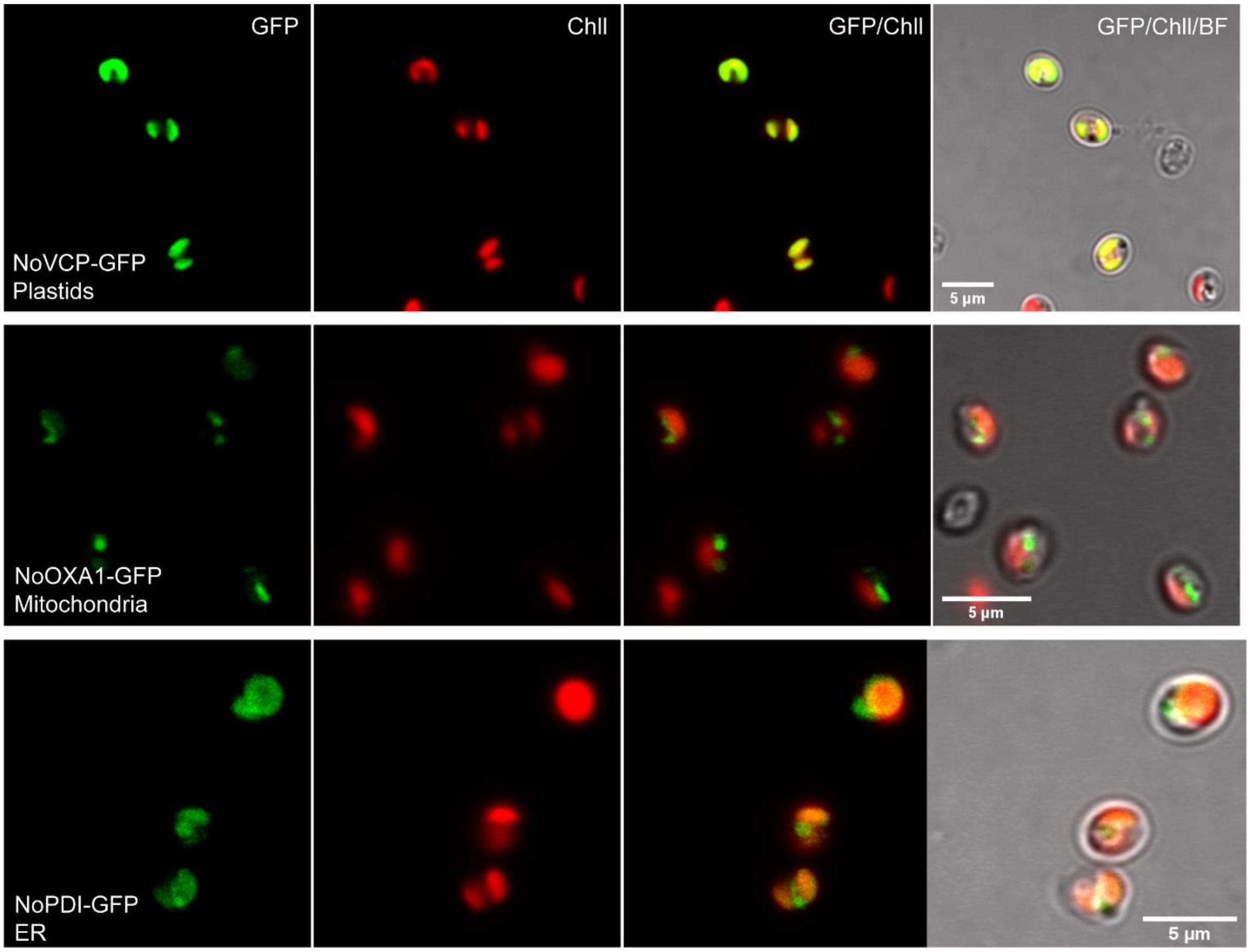

Figure S1. Localization studies of *N. oceanica* signal/transit peptides. NoVCP1, violaxanthin/chlorophyll a-binding protein 1; NoOxa1, mitochondrial oxidase assembly protein1; NoPDI, endoplasmic reticulum (ER) protein disulfide isomerase; GFP, green fluorescent protein; Chll, autofluorescence of chloroplast in red; BF, bright field.

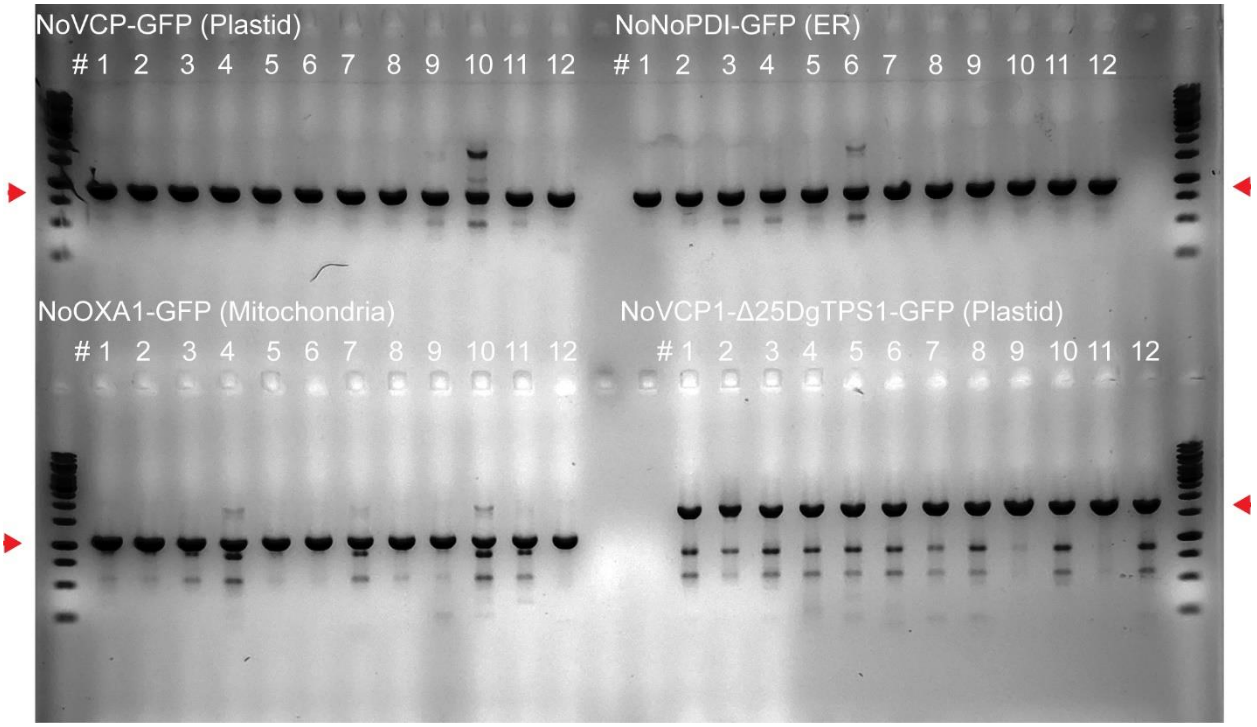

Figure S2. Colony PCR to screen the positive transformants. Arrowheads indicate the target PCR bands.

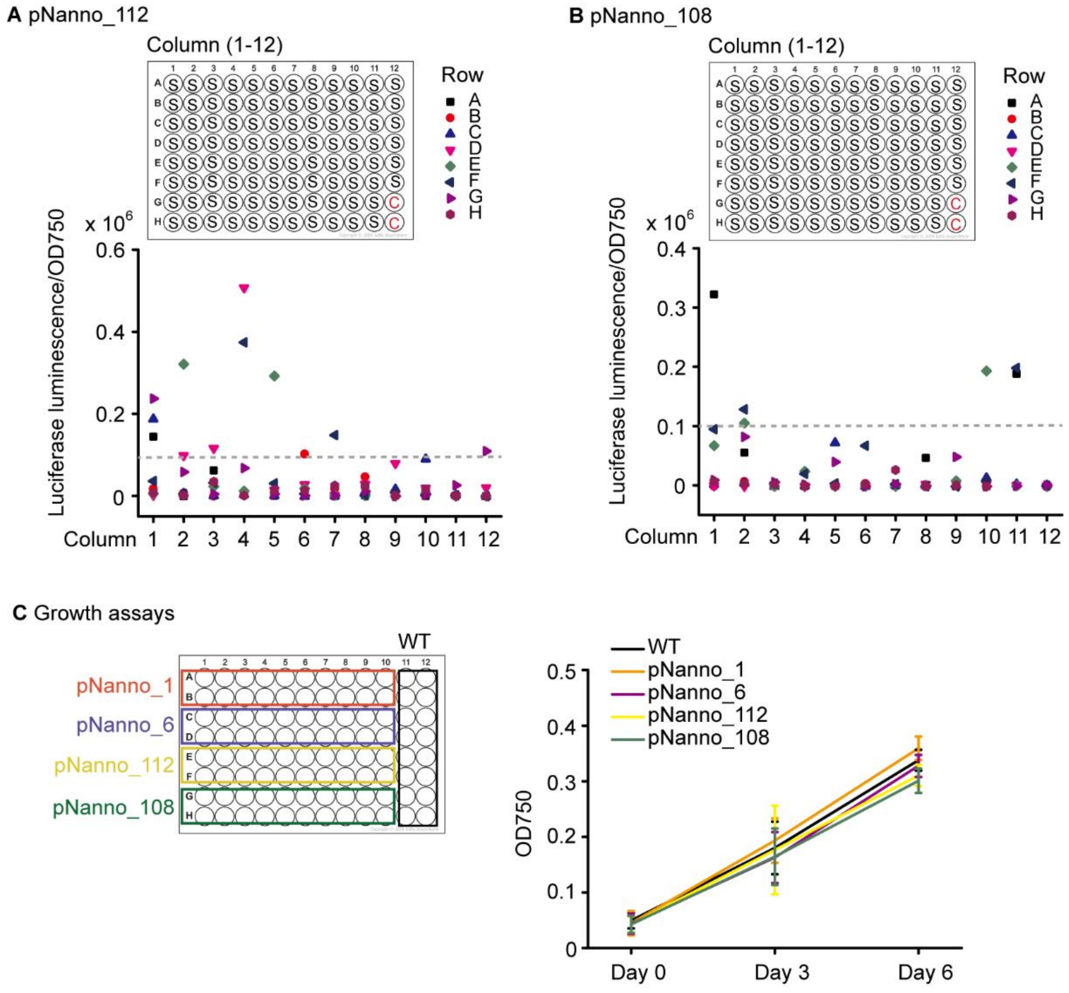

Figure S3. Mutant screening by the luminescence assays. *N. oceanica* cells of transformants (S) and controls (C) were grown and tested in 96 deep well plates, using NanoGlo substrate. A and B, Dot lines indicate the threshold (standardized luminescence, 1 x 10^5) for positive and productive transformants. Each well has an independent transformant, and the ones above the threshold were selected for further cultivation and analyses. C, Growth assays of the wild type (WT), vector control (pNanno_1), and transformants (pNanno_6, pNanno_112, and pNanno_108). No significance differences were observed in the growth rate of the samples with 3 biological replicates (three 96-well plates).

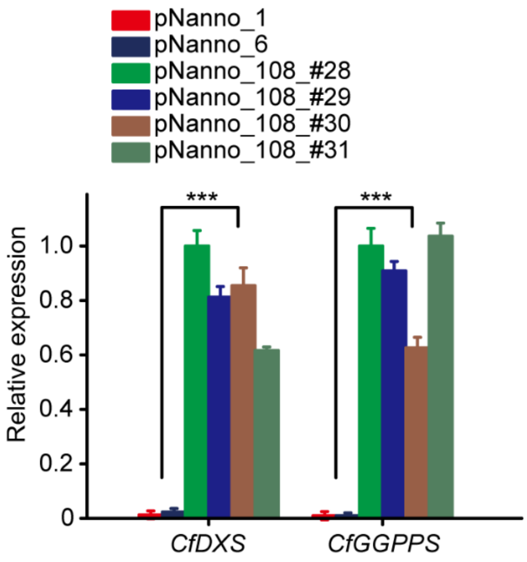

Figure S4. Asterisks indicate significant differences between the samples based on the *t*-test. ***, $p \leq 0.001$.

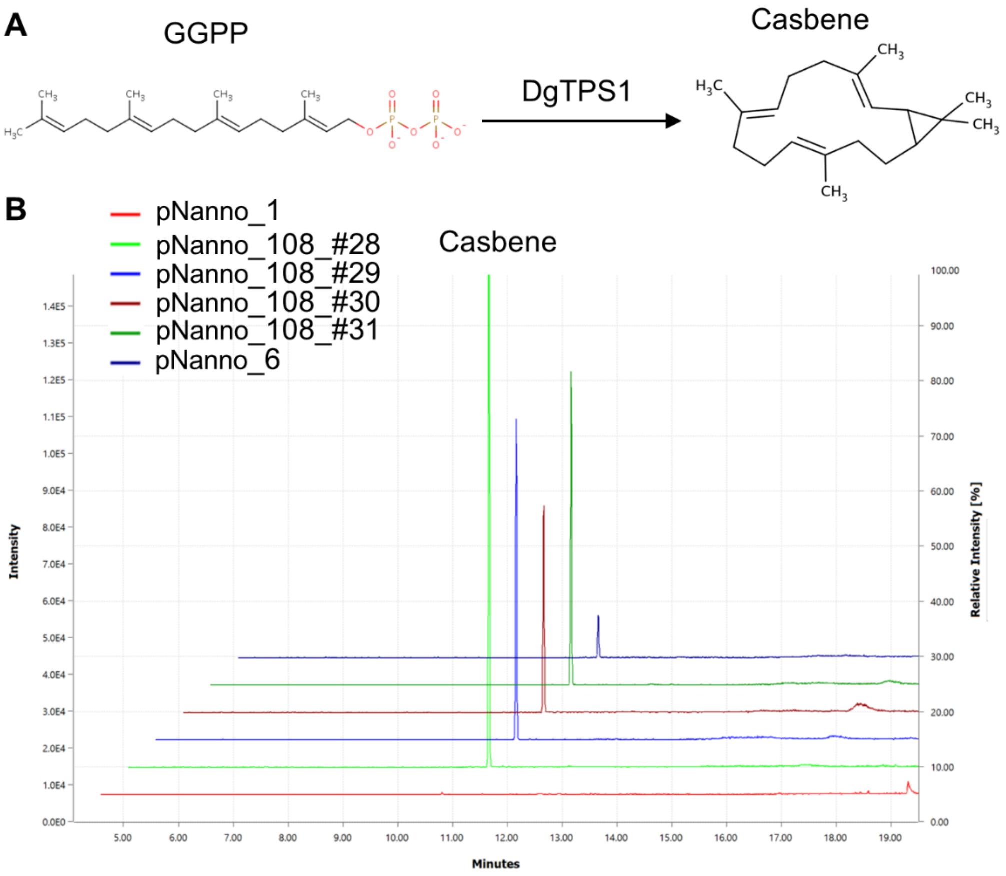

Figure S5. GC-MS analysis of casbene production in the *N. oceanica* transformants. A, Casbene produced by DgTPS1 (*Daphne genkwa* casbene synthase) with GGPP (geranylgeranyl diphosphate). B, Chromatograms of the pNanno_108 (Δ25_DgTPS1, Δ40_CfDXS, and Δ43_CfGGPPS) and pNanno_6 (Δ25DgTPS1-*C*-terminal eGFP) transformants with pNanno_1 (*C*-terminal eGFP) as the vector control.

2. Plasmid maps and sequences

pNOC-ARS-destiny-NeoR (10984 bp)

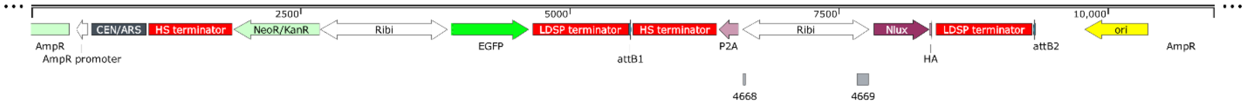

Sequence:

CGATCGTTGTCAGAAGTAAGTTGGCCGCAGTGTTATCACTCATGGTTATGGCAGCACTGCATAATTCTCTTACTGTCATGCCATCCGTAAGATGCTTTTCTGTGACTGGTGAGTACTCAACCAAGTCATTCTGAGAATAGTGTATGCGGCGACCGAGTTGCTCTTGCCCGGCG
TCAATACGGGATAATACCGCGCCACATAGCAGAACTTTAAAAGTGCTCATCATTGGAAAACGTTCTTCGGGGCGAAAACTCTCAAGGATCTTACCGCTGTTGAGATCCAGTTCGATGTAACCCACTCGTGCACCCAACTGATCTTCAGCATCTTTTACTTTCACCAGCGTTT
CTGGGTGAGCAAAAACAGGAAGGCAAAATGCCGCAAAAAAGGGAATAAGGGCGACACGGAAATGTTGAATACTCATACTCTTCCTTTTTCAATATTATTGAAGCATTTATCAGGGTTATTGTCTCATGAGCGGATACATATTTGAATGTATTTAGAAAAATAAACAAATA
GGGGTTCCGCGCACATTTCCCCGAAAAGTGCCACCTGGGTCCTTTTCATCACGTGCTATAAAAATAATTATAATTTAAATTTTTTAATATAAATATATAAATTAAAAATAGAAAGTAAAAAAAGAAATTAAAGAAAAAATAGTTTTTGTTTTCCGAAGATGTAAAAGACTCT
AGGGGGATCGCCAACAAATACTACCTTTTATCTTGTACTTCCTGCTCTCAGGTATTAATGCCGAATTGTTTCATCTTGTCTGTGTAGAAGACCACACACGAAAATCCTGTGATTTTACATTTTACTTATCGTTAATCGAATGTATATCTATTTAATCTGCTTTTCTTGTCTAATA
AATATATATGTAAAGTACGCTTTTTGTTGAAATTTTTTAAACCTTTGTTTATTTTTTTTTCTTCATTCCGTAACTCTTCTACCTTCTTTATTTACTTTCTAAAATCCAAATACAAAACATAAAAATAAATAAACACAGAGTAAATTCCCAAATTATTCCATCATTAAAAGATACG
AGGCGCGTGTAAGTTACAGGCAAGCGATCCGTCTAAGAAACCGGTtaaggcTAAtggatgggaagtcggtaagtgaaaagggctttgtgtaaggtggatgtcaaattttgccaacccgtgcctctgtgttcgcagtgtagcaaggaagcccacaccaccgcctcatattcaaatcgaaatcatcgaagaaatcggcgaggatctcatgccccaaagctgtcatcaaatgtgtcccaccacacgaag
atgccgagtaaatgacgtgagttgtcctatgattggcaggatgtggcatttaaagtccaaaaggccctgcttggtcggtcaccttttgctcgcctccccctcccgtcacaccctaaacacgaatgcagaagaaaatcatgtggggaagcaacgatttagaaatgggaatggaggctcttcacttcctgaatctgaccaagctggcggacacaaccgtagagggatctttgacctgcatcaatacaccgacatgttaataaatcgagtaccaaagaaaa
gctactgaagtcagtttcggattgtgttgtagaatcttttgagtcgtcactggaatgctatgctcgcttcaatcgtcgattagctttgtaaattatttctggtggcaaggcggggttcaagtagcaagtgggggcatggcagcacccctccgagcctcgttcgttcaggtgtttttgggacctttccctcccttcctgtcttcagacgcttttattttcctttccgttttctactagtgacaaaaaaagggcacacactctttactcgctcacaccctcgtactcggaca
cgcgctgttaccgactctGGTACCTCAAGCGCTGAAGAACTCGTCAAGAAGGCGATAGAAGGCGATGCGCTGCGAATCGGGAGCGGCGATACCGTAAAGCACGAGGAAGCGGTCAGCCCATTCGCCGCCAAGCTCTTCAGCAATATCACGGGTAGCCAACGCTATGTCCTGATAGC
GGTCCGCCACACCCAGCCGGCCACAGTCGATGAATCCAGAAAAGCGGCCATTTTCCACCATGATATTCGGCAAGCAGGCATCGCCATGGGTCACGACGAGATCCTCGCCGTCGGGCATGCTCGCCTTGAGCCTGGCGAACAGTTCGGCTGGCGCGAGCCCCTGATGCTCTT
CGTCCAGATCATCCTGATCGACAAGACCGGCTTCCATCCGAGTACGTGCTCGCTCGATGCGATGTTTCGCTTGGTGGTCGAATGGGCAGGTAGCCGGATCAAGCGTATGCAGCCGCCGCATTGCATCAGCCATGATGGATACTTTCTCGGCAGGAGCAAGGTGAGATGACA
GGAGATCCTGCCCCGGCACTTCGCCCAATAGCAGCCAGTCCCTTCCCGCTTCAGTGACAACGTCGAGCACAGCTGCGCAAGGAACGCCCGTCGTGGCCAGCCACGATAGCCGCGCTGCCTCGTCTTGCAGTTCATTCAGGGCACCGGACAGGTCGGTCTTGACAAAAAGA

[illegible]

pNanno_1 (6912 bp)

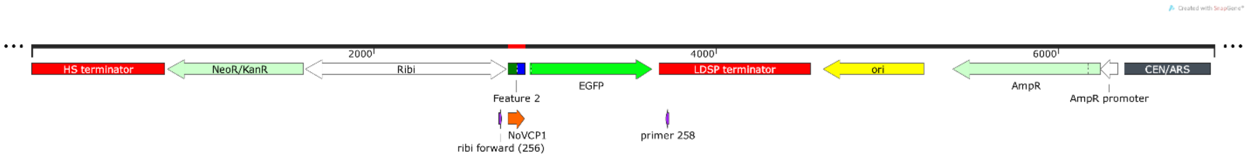

Sequence:

[illegible]

pNanno_6 (8664 bp)

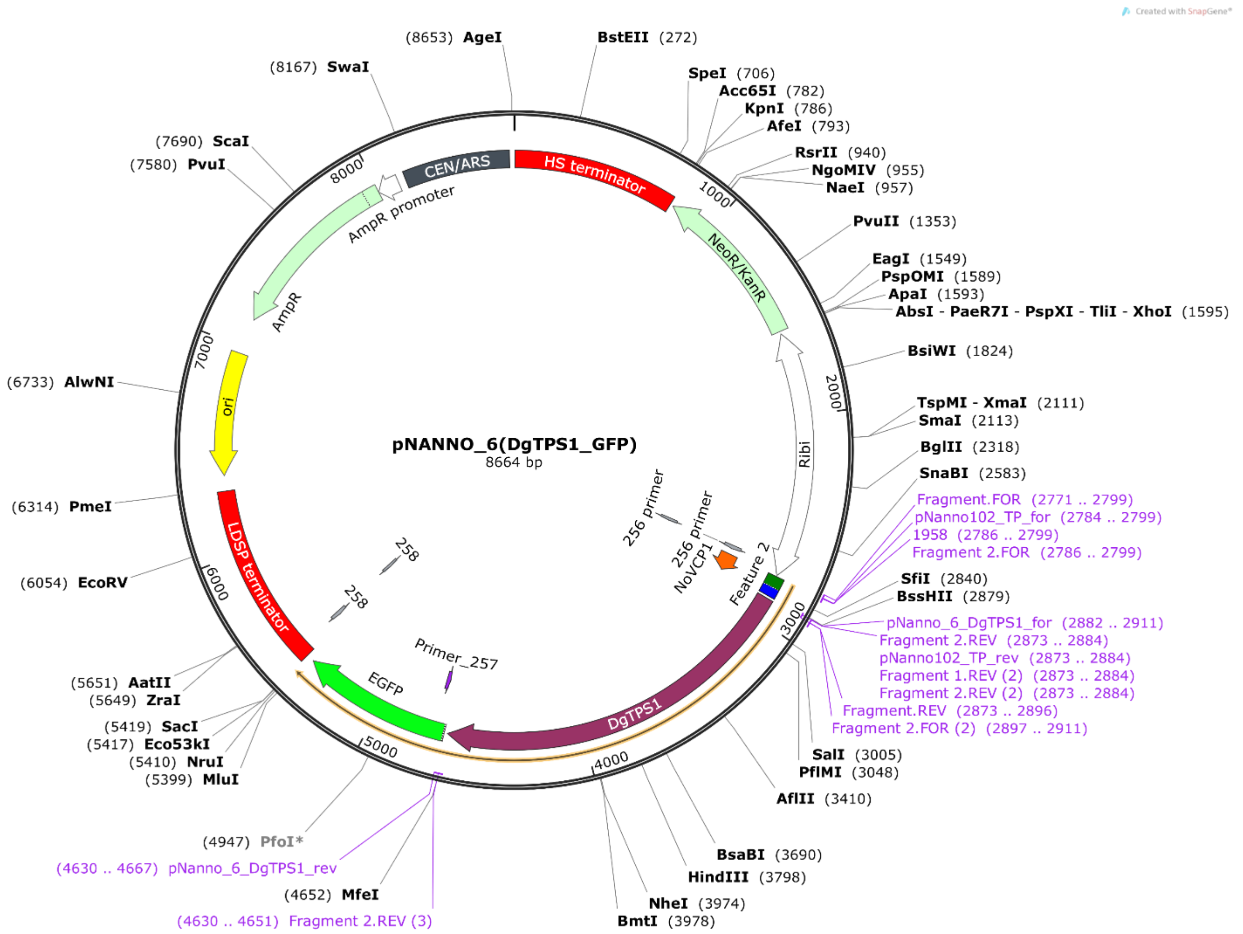

Sequence:

TAAtggatgggaagtcggtaagtgaaaagggctttgtgtaaggtggatgtcaaattttgccaacccgtgcctctgtgttcgcagtgtagcaaggaagcccacaccaccgcctcatattcaaatcgaaatcatcgaagaaatcggcgaggatctcatgccccaaagctgtcatcaaatgtgtcccaccacacgaagatgccgagtaaatgacgtgagttgtcctatgattggcaggatgtggcatttaaagtccaaaaggccctgcttggtcggtca
cctttgctcgcctcccctcccgtcacaccctaaacacgaatgcagaagaaaatcatgtggggaagcaacgatttagaaatgggaatggaggctcttcacttcctgaatctgaccaagctggcggacacaaccgtagagggatctttgacctgcatcaatacaccgacatgttaataaatcgagtaccaaagaaaagctactgaagtcagtttcggattgtgttgtagaatcttttgagtcgtcactggaatgctatgctcgcttcaatcgtcgattagctt
tgtaaattatttctggtggcaaggcggggttcaagtagcaagtgggggcatggcagcacccctccgagcctcgttcgttcaggtgtttttgggacctttccctcccttcctgtcttcagacgcttttattttcctttccgttttctactagtgacaaaaaaagggcacacactctttactcgctcacaccctcgtactcggacacgcgctgttaccgactctGGTACCTCAAGCGCTGAAGAACTCGTCAAGAAGGCGATA
GAAGGCGATGCGCTGCGAATCGGGAGCGGCGATACCGTAAAGCACGAGGAAGCGGTCAGCCCATTCGCCGCCAAGCTCTTCAGCAATATCACGGGTAGCCAACGCTATGTCCTGATAGCGGTCCGCCACACCCAGCCGGCCACAGTCGATGAATCCAGAAAAGCGGCCA
TTTTCCACCATGATATTCGGCAAGCAGGCATCGCCATGGGTCACGACGAGATCCTCGCCGTCGGGCATGCTCGCCTTGAGCCTGGCGAACAGTTCGGCTGGCGCGAGCCCCTGATGCTCTTCGTCCAGATCATCCTGATCGACAAGACCGGCTTCCATCCGAGTACGTGCTC
GCTCGATGCGATGTTTCGCTTGGTGGTCGAATGGGCAGGTAGCCGGATCAAGCGTATGCAGCCGCCGCATTGCATCAGCCATGATGGATACTTTCTCGGCAGGAGCAAGGTGAGATGACAGGAGATCCTGCCCCGGCACTTCGCCCAATAGCAGCCAGTCCCTTCCCGCTT
CAGTGACAACGTCGAGCACAGCTGCGCAAGGAACGCCCGTCGTGGCCAGCCACGATAGCCGCGCTGCCTCGTCTTGCAGTTCATTCAGGGCACCGGACAGGTCGGTCTTGACAAAAAGAACCGGGCGCCCCTGCGCTGACAGCCGGAACACGGCGGCATCAGAGCAGCC
GATTGTCTGTTGTGCCCAGTCATAGCCGAATAGCCTCTCCACCCAAGCGGCCGGAGAACCTGCGTGCAATCCATCTTGTTCAATCATGGGCCCCTCGAGggttgcgtgtgtatctgtgtgcagtggatattgttaccgagtttggtgagcgtgagtccgttagtgccctggtggtggtggattaggagagtgggtgactcggtgtccatggctttcttcg
ctcattataggaggggaaaggaatgagggagggtggggagaccgcgtctgttgttgaccaccgatttacttcttgcctcccttcccctccctcccctcaatccgtacgacacaaatagtagccgagtgtctgctgcagagcgcatgattagtgtggtagacaacgagggagggaaggatgtacagggcatggcacggagaagcgatggtggccaggaagaggagaggtcgTgagaacaggatgtgttgcgaatggataaaaacagaaagc
gatggctctgggcttcgaaagcaggggacattaggacgtgtagaccatctcgacggatccctctgtatctctgttgtgcgtgaatgttttctgtgcacgtgtagtgtgtgagagtagaacccgggaactcgaacagagaaaagcatgggtggctgtggtgtggaggcttcgttcccaccacatgcccttctccttcgcctcgcctctccctgccttcttccacgcacccttgcgcccctcgttctcaatacctggctcacttccaccattcaaacaaccatc
acgatacaggcatttatctatcgttgaagacttcttcctccggtagatcttagccaaggtaagaagaggggcatgcagcaaggagaaagaaatgatgcatgatgaggaatagaaggggaggagggagggatatgatgggaagcgaaagcgcatattctggtggtctgctgcctgatggggagtctagctgtgacactgaggacggtggctgctggtggctgcgggcgctgccttggtgatcaatgggagtaaagggagggaaggaggtagc
gtgaacggatgacgcggagaagtttaggggtctctttacgtatcgcccctgccgcccgcctctctgcgataaatgtgcctgttaccctgcagcctctattcttcactgtgttcctgttttccaacagcctctattcttccctgtcttttgttgcagtggcgtcatcctctttgccccagtcgtcgttctctcgactcactcactccccccctccttccctccctccatccacagaatcgATGAAGACCGCCGCTCTCCTCACTGTCTCC
ACCCTCATGGGCGCCCAGGCCTTTATGGCCCCCGCCCCCAAGTTCTCCCGCACCCGCGGTGTTGCGCGCCGCGCCctcGTTGCTGCTGCTGTGTCCGAGTTACAGAACAGCAAATGCTGCTTCTCACAATATTCCCTCGTCTTAGGAACAAGATCGGTTCCAGGTCCGGCTCCT
CTTCATGCCATAGCCGTCGACACCCAGCCTCGTCGATCTGCTGATTACACCCCCAACATCTGGGGCAACCATTTCATTGCCACCTCTCCAACTGCTTTGGGCATTGAAAGTGAAGTGGAGGAGCATGAAAAGCTGAAGCAAGAAGTGAGGGGCGTGATAGCCGATGCAATG
GAAGATCCAAACAAGAACCTGGACTTGATTGACGCAATCCAAAGGTTAGGGGTGTCGTATCATTTCGAAGAGGAAGTTGAGGAGTTTTTGCAGAAAACGCACGCCAATCTCCATGGATTCATCAAGGATAATGCAGACAATCTTCAACAAATCTCATTATGCTTTCGGTTA
CTGAGGCAACATGGCTACCAAGTTTCATGCAATATGTTCACCAACTTCATAGAAGATGATGGGAGCTTCAGGGGATCTCTTAAGCAAGACTTTGAAGGACTGATGAGTCTTTATGAAGCTGCACATCTGAGGGTTCATGGAGAAGAAATTCTCGAAAACGCCCTCGCTTTT
AGTACCACCCAACTCAAACTCATCGCAAAAGCTGGCTCCAAATTTTCTGCAATTATCAATCACAGTTTACGGAATCCAATTCGGAAGAACTTCCCCAGGCTTGACACAAGGTTCTACATCTCTGTCTATCAAGAGATCAAGCCTAACTCACATAACCCATATTTGTTACAAT
TTTCCAAGTTGGATTTTAATCTTGTACAGAAGCAGTACCGAGAGGAGCTGAATCAAATATCAAGAtGGTGGAATGAGCTGGATGCTGAAACGAAgTTCCCGTTTGCAAgAAACAGGAttgtAGAAGCTTTCTTCTTCATTATGGGAACTTATTTTGAACCCCGATACAGATTGG
GTAGAAAGATAGCCACAAAAGCTCTAGCAATGTTGACCATTCTTGACGACATCTATGATGTCCATGGCACGCcTCAAGAACTCGACCTTCTTAcTCAGGCATTGGAGCGGTGGGATCCCAACTTGGCTAGCGAGATGCCAGAGTACATAAAAAATTTCTATGTGGCACTTTT
GGACACATTCAAGGATTTGGAGGAGGATATGGGTACTGCACAAGGTGCTGATGATCATGTTGCTGATAAGATAAAGTATTGTAAAGAAGGGTTGATAAAGGTGGCTAAAGCCTACCACACTGAAGCCAAATGGTATGAGCAGAATTACACACCCACCATGGAGGAGTAC
ATGGAGGTTGCCCTTGTGAGTGTGGCCACACCATTCATAGGAGCCATGGTGTTGCTTGGGATGGGACACCTTGCAAACAAAGATGCTTTTGAATGGTTTCGCACGCATCCCAAGATTGTGGCATCAGCTTGTCTAATTCCAAGGCTTCAAGATGACATTGTGGGCCATGAGT
TTGAGCAAAAGAGGGGGCATTCGGCTTCATGCGTGGACTGCTACGTGAAGGAGCATGGCAAGGGGAAAGAAGAAGCGGTGGAGGCATTTTACAAGCAAGCGAGCGATGCATGGAAAGATGCAAACGCTGATTTCATTCGGCCAACCGCTGTTCCCATGCAAGTTCTCAC
GACGCTCCTCAACATCTCCCGCATAGCACACGTTGTGTACAACGACTGCGATGGATACGCTGATCCTCATGCTATCAAAGCTCTAGTCGCTTCCGTCTTCGTCCACCCAATTCCTTATAACCAATTGgggagcggcatggtgagcaagggcgaggagctgttcaccggggtggtgcccatcctggtcgagctggacg
gcgacgtaaacggccacaagttcagcgtgtccggcgagggcgagggcgatgccacctacggcaagctgaccctgaagttcatctgcaccaccggcaagctgcccgtgccctggcccaccctcgtgaccaccttcacctacggcgtgcagtgcttcagccgctaccccgaccacatgaagcagcacgacttcttcaagtccgccatgcccgaaggctacgtccaggagcgcaccatcttcttcaaggacgacggcaactacaagacccgcg
ccgaggtgaagttcgagggcgacaccctggtgaaccgcatcgagctgaagggcatcgacttcaaggaggacggcaacatcctggggcacaagctggagtacaactacaacagccacaacgtctatatcatggccgacaagcagaagaacggcatcaaggtgaacttcaagatccgccacaacatcgaggacggcagcgtgcagctcgccgaccactaccagcagaacacccccatcggcgacggccccgtgctgctgcccgacaac
cactacctgagcacccagtccgccctgagcaaagaccccaacgagaagcgcgatcacatggtcctgctggagttcgtgaccgccgccgggatcactcacggcatggacgagctgtacaagggaagcggcaggcctacgcgtgagtcgcgaTGAGCTCgaaagatccaagagagacgagtagagatttttttttgggattgatgtttgtcgttctttgagttgtcgtcgagttacgccttttgtaagaatgttccgcaggagaggaggagg
atgggcatgagtgagggtgagagggcttgcccgcttttttttttaaaaacgctgctcttcgtggttgtcaaacaaacccccccatagaaacgattttgttacggtgcggtccagacgtcacttgaatggctccgcggaaaggccagggagggaagggggggagggaggaaacatgaaacatgttgaacggctcaacagggtttgggggacaagagaggtagcgccctgatggactgctccctcccctcctttccctcaatgtctcattcatccatgctt
ccccttctctctctcccctccgttccatcccccgcgggcgtggtagtggcgtgatgggatccactaaaatgtacgtgtaagaaaagccggtgagcttacgcttttgtgaaagtgggagtacgagtgttgtgtgtgtgtgtagtggtttcagaccccagacagaggcgaagcagaaaaagcagacgatgaagacgacgaagaaatgagcagtctatttttatcgtggaaacagaagaggtgatatcgtctcgttctttgttatcacctaccccgcgtgc
atgtacatgcagccttttttattttgtaatctttcccgaaaaatcaaccgccacctcccccccgccttctctcacccatcatcttctcctgtttatcttctactttacactagatcgcatggcacatctccctcgcaatccatcggtgcaaccatcatcgatcccactcctccctccctccctccctccctcctcgcatctcttctaagaaatccgctagTGTTTAAACATGTGAGCAAAAGGCCAGCAAAAGGCCAGGAAC
CGTAAAAAGGCCGCGTTGCTGGCGTTTTTCCATAGGCTCCGCCCCCCTGACGAGCATCACAAAAATCGACGCTCAAGTCAGAGGTGGCGAAACCCGACAGGACTATAAAGATACCAGGCGTTTCCCCCTGGAAGCTCCCTCGTGCGCTCTCCTGTTCCGACCCTGCCGCTT
ACCGGATACCTGTCCGCCTTTCTCCCTTCGGGAAGCGTGGCGCTTTCTCATAGCTCACGCTGTAGGTATCTCAGTTCGGTGTAGGTCGTTCGCTCCAAGCTGGGCTGTGTGCACGAACCCCCCGTTCAGCCCGACCGCTGCGCCTTATCCGGTAACTATCGTCTTGAGTCCAA
CCCGGTAAGACACGACTTATCGCCACTGGCAGCAGCCACTGGTAACAGGATTAGCAGAGCGAGGTATGTAGGCGGTGCTACAGAGTTCTTGAAGTGGTGGCCTAACTACGGCTACACTAGAAGAACAGTATTTGGTATCTGCGCTCTGCTGAAGCCAGTTACCTTCGGAA
AAAGAGTTGGTAGCTCTTGATCCGGCAAACAAACCACCGCTGGTAGCGGTGGTTTTTTTGTTTGCAAGCAGCAGATTACGCGCAGAAAAAAAGGATCTCAAGAAGATCCTTTGATCTTTTCTACGGGGTCTGACGCTCAGTGGAACGAAAACTCACGTTAAGGGATTTTGG
TCATGAGATTATCAAAAAGGATCTTCACCTAGATCCTTTTAAATTAAAAATGAAGTTTTAAATCAATCTAAAGTATATATGAGTAAACTTGGTCTGACAGTTACCAATGCTTAATCAGTGAGGCACCTATCTCAGCGATCTGTCTATTTCGTTCATCCATAGTTGCCTGACTC
CCCGTCGTGTAGATAACTACGATACGGGAGGGCTTACCATCTGGCCCCAGTGCTGCAATGATACCGCGAGACCCACGCTCACCGGCTCCAGATTTATCAGCAATAAACCAGCCAGCCGGAAGGGCCGAGCGCAGAAGTGGTCCTGCAACTTTATCCGCCTCCATCCAGTCT
ATTAATTGTTGCCGGGAAGCTAGAGTAAGTAGTTCGCCAGTTAATAGTTTGCGCAACGTTGTTGCCATTGCTACAGGCATCGTGGTGTCACGCTCGTCGTTTGGTATGGCTTCATTCAGCTCCGGTTCCCAACGATCAAGGCGAGTTACATGATCCCCCATGTTGTGCAAAA
AAGCGGTTAGCTCCTTCGGTCCTCCGATCGTTGTCAGAAGTAAGTTGGCCGCAGTGTTATCACTCATGGTTATGGCAGCACTGCATAATTCTCTTACTGTCATGCCATCCGTAAGATGCTTTTCTGTGACTGGTGAGTACTCAACCAAGTCATTCTGAGAATAGTGTATGCGG
CGACCGAGTTGCTCTTGCCCGGCGTCAATACGGGATAATACCGCGCCACATAGCAGAACTTTAAAAGTGCTCATCATTGGAAAACGTTCTTCGGGGCGAAAACTCTCAAGGATCTTACCGCTGTTGAGATCCAGTTCGATGTAACCCACTCGTGCACCCAACTGATCTTCAG
CATCTTTTACTTTCACCAGCGTTTCTGGGTGAGCAAAAACAGGAAGGCAAAATGCCGCAAAAAAGGGAATAAGGGCGACACGGAAATGTTGAATACTCATACTCTTCCTTTTTCAATATTATTGAAGCATTTATCAGGGTTATTGTCTCATGAGCGGATACATATTTGAATG
TATTTAGAAAAATAAACAAATAGGGGTTCCGCGCACATTTCCCCGAAAAGTGCCACCTGGGTCCTTTTCATCACGTGCTATAAAAAATAATTATAATTTAAATTTTTTAATATAAATATATAAATTAAAAATAGAAAGTAAAAAAAGAAATTAAAGAAAAAATAGTTTTTGT
TTTCCGAAGATGTAAAAGACTCTAGGGGGATCGCCAACAAATACTACCTTTTATCTTGTACTTCCTGCTCTCAGGTATTAATGCCGAATTGTTTCATCTTGTCTGTGTAGAAGACCACACACGAAAATCCTGTGATTTTACATTTTACTTATCGTTAATCGAATGTATATCTAT
TTAATCTGCTTTTCTTGTCTAATAAATATATATGTAAAGTACGCTTTTTGTTGAAATTTTTTAAACCTTTGTTTATTTTTTTTTCTTCATTCCGTAACTCTTCTACCTTCTTTATTTACTTTCTAAAATCCAAATACAAAACATAAAAATAAATAAACACAGAGTAAATTCCCAA
ATTATTCCATCATTAAAAGATACGAGGCGCGTGTAAGTTACAGGCAAGCGATCCGTCTAAGAAACCGGTtaaggc

pNanno_112 (12062 bp)

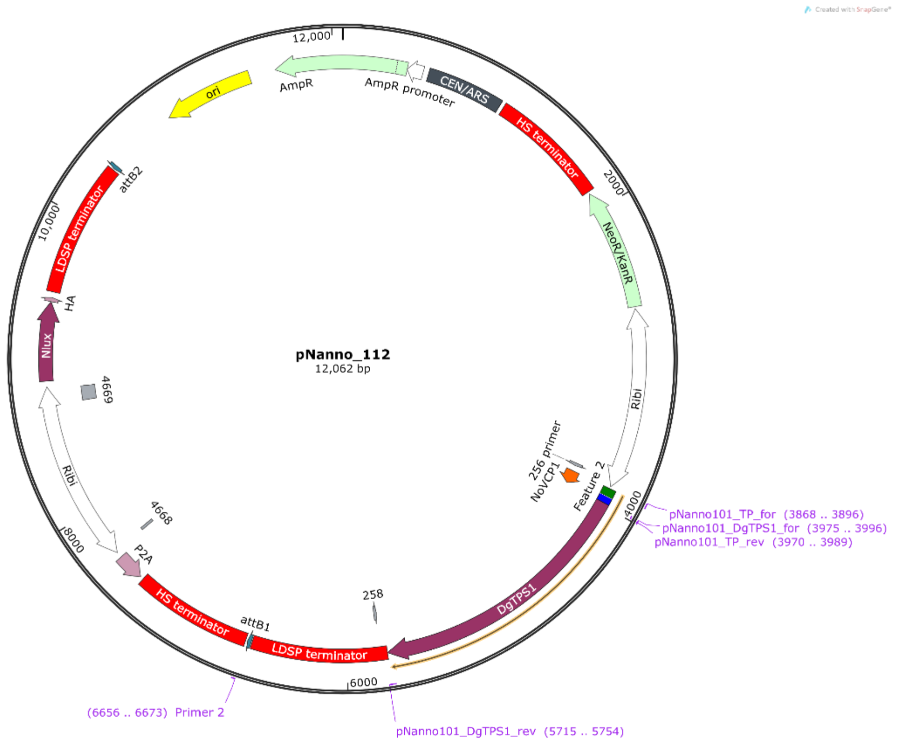

Sequence:

[illegible]

pNanno_108 (15257 bp)

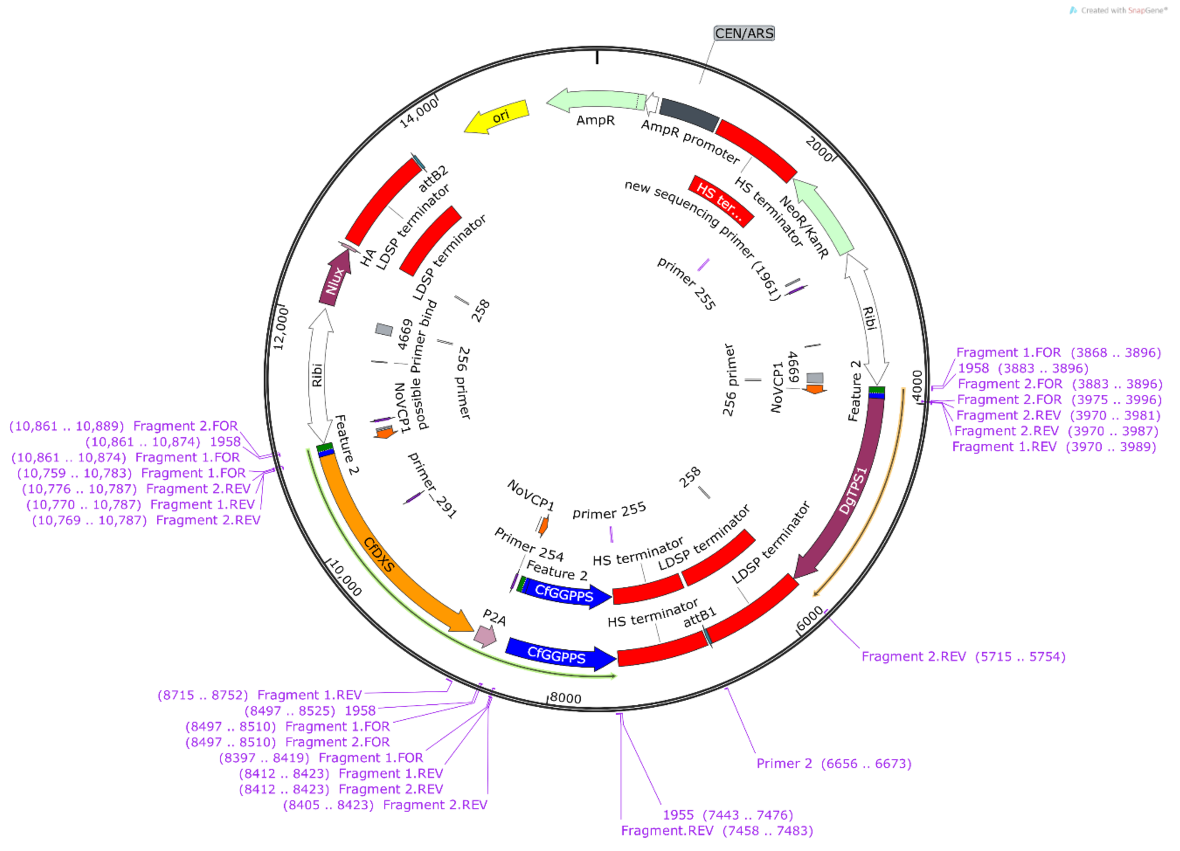

pNanno108(pNOC_ARS_DEST_NeoR_TP▲DgTPS1_TP_▲DXS_2A_TP_▲GGPPS)
15,257 bp

Sequence:

[illegible]

agcgtgagtccgttagtgccctggtggtggtggattaggagagtgggtgactcggtgtccatggctttcttcgctcattataggaggggaaaggaatgagggagggtggggagaccgcgtctgttgttgaccaccgatttacttcttgcctcccttcccctccctcccctcaatccgtacgacacaaatagtagccgagtgtctgctgcagagcgcatgattagtgtggtagacaacgagggagggaaggatgtacagggcatggcacggagaag
cgatggtggccaggaagaggagaggtcgTgagaacaggatgtgttgcgaatggataaaaacagaaagcgatggctctgggcttcgaaagcaggggacattaggacgtgtagaccatctcgacggatccctctgtatctctgttgtgcgtgaatgttttctgtgcacgtgtagtgtgtgagagtagaacccgggaactcgaacagagaaaagcatgggtggctgtggtgtggaggcttcgttcccaccacatgcccttctccttcgcctcgcctctc
cctgccttcttccacgcacccttgcgcccctcgttctcaatacctggctcacttccaccattcaaacaaccatcacgatacaggcatttatctatcgttgaagacttcttcctccggtagatcttagccaaggtaagaagaggggcatgcagcaaggagaaagaaatgatgcatgatgaggaatagaaggggaggagggagggatatgatgggaagcgaaagcgcatattctggtggtctgctgcctgatggggagtctagctgtgacactgaggac
ggtggctgctggtggctgcgggcgctgccttggtgatcaatgggagtaaagggagggaaggaggtagcgtgaacggatgacgcggagaagtttaggggtctctttacgtatcgcccctgccgcccgcctctctgcgataaatgtgcctgttaccctgcagcctctattcttcactgtgttcctgttttccaacagcctctattcttccctgtcttttgttgcagtggcgtcatcctctctttgccccagtcgtcgttctctcgactcactcactccccccctcct
tccctccctccatccacagaatcgatTGGCGCGCCctcGTTAACCAATTGgggagcggcATGGTGTTTACTCTGGAGGACTTCGTGGGCGACTGGCGGCAGACGGCTGGCTATAACCTCGACCAGGTGCTGGAGCAGGGCGGTGTGTCCTCGCTTTTCCAGAACCTGGGCGTCTCCGTGACCCCAA
TCCAGCGCATCGTCCTGAGCGGCGAGAACGGCCTGAAGATCGACATCCACGTCATCATTCCCTACGAGGGCCTGAGCGGGGACCAAATGGGCCAGATCGAGAAGATCTTCAAGGTTGTGTACCCCGTGGACGATCACCACTTCAAGGTGATACTGCACTACGGGACGCTG
GTCATCGACGGTGTGACGCCGAACATGATTGACTACTTCGGCCGTCCTTACGAGGGCATTGCCGTGTTCGACGGCAAGAAGATCACGGTGACCGGCACCCTGTGGAACGGCAATAAGATCATTGACGAGCGGCTGATCAACCCGGATGGCTCGCTGTTGTTCCGCGTGACC
ATCAACGGGGTAACCGGCTGGCGCCTGTGCGAGCGCATCCTGGCCGGATACCCCTACGACGTGCCCGACTACGCGggaagcggcaggcctacgcgtgagtcgcgaTGAGCTCgaaagatccaagagagacgagtagagatttttttttgggattgatgtttgtcgttctttgagttgtcgtcgagttacgccttttgtaagaatgttccgcaggagaggaggaggat
gggcatgagtgagggtgagagggcttgcccgcttttttttttaaaaacgctgctcttcgtggttgtcaaacaaaccccccatagaaacgattttgttacggtgcggtccagacgtcacttgaatggctccgcggaaaggccagggagggaagggggagggaggaaacatgaaacatgttgaacggctcaacagggtttgggggacaagagaggtagcgccctgatggactgctccctcccctcctttccctcaatgtctcattcatccatgcttcc
cccttctctctctcccctccgttccatccccgcgggcgtggtagtggcgtgatgggatccactaaaatgtacgtgtaagaaaagccggtgagcttacgcttttgtgaaagtgggagtacgagtgttgtgtgtgtgtgtagtggtttcagaccccagacagaggcgaagcagaaaaagcagacgatgaagacgacgaagaaatgagcagtctatttttatcgtggaaacagaagaggtgatatcgtctcgttctttgttatcacctaccccgcgtgcatg
tacatgcagccttttatttgtaatctttcccgaaaaatcaaccgccacctccccccgccttctctcacccatcatcttctcctgtttatcttctactttacactagatcgcatggcacatctccctcgcaatccatcggtgcaaccatcatcgatcccactcctccctccctccctccctcctcgcatctcttctaagaaatccgctAGAGCGCCGACCCAGCTTTCTTGTACAAAGTGGTCTTAAGATTGAA
TCCTGTTGCCGGTCTTGCGATGATTATCATATAATTTCTGTTGAATTACGTTAAGCATGTAATAATTAACATGTAATGCATGACGTTATTTATGAGATGGGTTTTTATGATTAGAGTCCCGCAATTATACATTTAATACGCGATAGAAAACAAAATATAGCGCGCAAACTAGG
ATAAATTATCGCGCGCGGTGTCATCTATGTTACTAGGCTGCATTAATGAATCGGCCAACGCGCGGGGAGAGGCGGTTTGCGTATTGGGCGCTCTTCCGCTTCCTCGCTCACTGACTCGCTGCGCTCGGTCGTTCGGCTGCGGCGAGCGGTATCAGCTCACTCAAAGGCGGTA
ATACGGTTATCCACAGAATCAGGGGATAACGCAGGAAAGAACATGTGAGCAAAAGGCCAGCAAAAGGCCAGGAACCGTAAAAAGGCCGCGTTGCTGGCGTTTTTCCATAGGCTCCGCCCCCCTGACGAGCATCACAAAAATCGACGCTCAAGTCAGAGGTGGCGAAACC
CGACAGGACTATAAAGATACCAGGCGTTTCCCCCTGGAAGCTCCCTCGTGCGCTCTCCTGTTCCGACCCTGCCGCTTACCGGATACCTGTCCGCCTTTCTCCCTTCGGGAAGCGTGGCGCTTTCTCATAGCTCACGCTGTAGGTATCTCAGTTCGGTGTAGGTCGTTCGCTCC
AAGCTGGGCTGTGTGCACGAACCCCCCGTTCAGCCCGACCGCTGCGCCTTATCCGGTAACTATCGTCTTGAGTCCAACCCGGTAAGACACGACTTATCGCCACTGGCAGCAGCCACTGGTAACAGGATTAGCAGAGCGAGGTATGTAGGCGGTGCTACAGAGTTCTTGAAG
TGGTGGCCTAACTACGGCTACACTAGAAGAACAGTATTTGGTATCTGCGCTCTGCTGAAGCCAGTTACCTTCGGAAAAAGAGTTGGTAGCTCTTGATCCGGCAAACAAACCACCGCTGGTAGCGGTGGTTTTTTTGTTTGCAAGCAGCAGATTACGCGCAGAAAAAAAGGA
TCTCAAGAAGATCCTTTGATCTTTTCTACGGGGTCTGACGCTCAGTGGAACGAAAACTCACGTTAAGGGATTTTGGTCATGAGATTATCAAAAAGGATCTTCACCTAGATCCTTTTAAATTAAAAATGAAGTTTTAAATCAATCTAAAGTATATATGAGTAAACTTGGTCTG
ACAGTTACCAATGCTTAATCAGTGAGGCACCTATCTCAGCGATCTGTCTATTTCGTTCATCCATAGTTGCCTGACTCCCCGTCGTGTAGATAACTACGATACGGGAGGGCTTACCATCTGGCCCCAGTGCTGCAATGATACCGCGAGACCCACGCTCACCGGCTCCAGATTT
ATCAGCAATAAACCAGCCAGCCGGAAGGGCCGAGCGCAGAAGTGGTCCTGCAACTTTATCCGCCTCCATCCAGTCTATTAATTGTTGCCGGGAAGCTAGAGTAAGTAGTTCGCCAGTTAATAGTTTGCGCAACGTTGTTGCCATTGCTACAGGCATCGTGGTGTCACGCTC
GTCGTTTGGTATGGCTTCATTCAGCTCCGGTTCCCAACGATCAAGGCGAGTTACATGATCCCCCATGTTGTGCAAAAAAGCGGTTAGCTCCTTCGGTCCTC

3. Table S1 Primer sequences

Primer ID	Primer Name	Sequence	Uses	Orentation
1936	pNanno_6_DgTPS1_for	CGCCGCGCCctcGTTGCTGCTGCTGTGTCC	amplification of ▲DgTPS1 for cloning inframe of GFP in pNanno1	Forward
1937	pNanno_6_DgTPS1_rev	tgccgctcccCAATTGGTTATAAGGAATTGGGTGGACG		Reverse
1940	pNanno101/102_TP_for	gaatcgatTGGCGCGATGAAGACCGCCGC	amplification of transit peptide for three fragment cloning with ▲DgTPS1 into pNOC-ARS-destiny-NeoR	Forward
1941	pNanno101_TP_rev	GCAGCAGCGCGCGCAACACC		Reverse
1942	pNanno101_DgTPS1_for	TGCGCGCGCTGCTGCTGTGTCC	amplification of ▲DgTPS1 for three fragment cloning with transit peptide into pNOC-ARS-destiny-NeoR	Forward
1943	pNanno101_DgTPS1_rev	ctcttggatctttcGTTAGTTATAAGGAATTGGGTGGACG		Reverse
1947	pNanno103_DgTPS1_for	CctcGTTAACCAATTGatgGCTGCTGCTGTGTCCGA	amplification of ▲DgTPS1 for cloning into pNOC-ARS-destiny-NeoR	Forward

1948	pNanno103_DgTPS1_rev	ctttcGAGCTCAtcgGTTATAAGGAATTGGGTGGACG		Reverse
1951	pNanno105_DXS_rev	CACCCTAGGGCGGCCCATGTTGATCAAATGAAGACTGT	amplification of ▲CfDXS for three fragment cloning with transit peptide into pNOC-stacked-MCS-Nlux	Reverse
1952	pNanno105_DXS_for	TTGCGCGCGCTGCTCTGTACCAGGA		Forward
1953	pNanno105_TP_rev	GAGCAGCGCGCGCAACACC	amplification of transit peptide for three fragment cloning with ▲CfDXS into pNOC-stacked-MCS-Nlux	Reverse
1954	pNanno105_TP_for	acacgcaaccCTCGAATGAAGACCGCCGC		Forward
1955	pNanno_106_GGPPS_rev	tctGGTACCTCAAGCCTAGTTCTGCCTGTGAGCA	amplification of ▲CfGGPPS for three fragment cloning with transit peptide into pNOC-stacked-MCS-Nlux	Reverse
1956	pNanno_106_GGPPS_for	TTGCGCGCTCCTTCGCCGTCTCC		Forward
1957	pNanno106_TP_rev	CGAAGGAGCGCGCAACACC	amplification of transit peptide for three fragment cloning with ▲CfGGPPS into pNOC-stacked-MCS-Nlux	Reverse
1958	pNanno106_TP_for	CCGGGGCCCTCTAGAAGCATGAAGACCGCCGCTCT		Forward
1959	pNanno_107_GGPPS_rev	tctGGTACCTCAAGCGCTGTTCTGCCTGTGAGCAAT	amplification of ▲CfGGPPS for cloning into pNOC-stacked-MCS-Nlux	Reverse
1960	pNanno_107_GGPPS_for	GGGCCCTCTAGAAGCTCCTTCGCCGTCTCC		Forward

254	pNanno_108_CPCR_rev	GCAAGGAGAAGTTCGTCGCCC	Colony PCR	Reverse
255	pNanno_108_CPCR_for	CACTCTTTACTCGCTCACACC	Colony PCR	Forward
256	pNanno_6/112_CPCR_for	TTCTCTCGACTCACTCACTCC	Colony PCR	Forward
257	pNanno_6_CPCR_rev	GTGCCCATCCTGGTCGAGCTG	Colony PCR	Reverse
258	pNanno_112_CPCR_rev	GGGATTGATGTTTGTCGTTCT	Colony PCR	Reverse
CfDXS_for	CfDXS_for	CAAAATCCTTCCCTTCCCAAC	qRT-PCR	Forward
CfDXS_rev	CfDXS_rev	ATGATGGACAGAGCTGGAC		Reverse
CfGGPPS_for	CfGGPPS_for	TGCAATGCAAATCCACCAC	qRT-PCR	Forward
CfGGPPS_rev	CfGGPPS_rev	AAGCCCACCAATCACAAAG		Reverse
NoACTIN_for	NoACTIN_for	GCCGTTATTGGATGGATATG	qRT-PCR	Forward
NoACTIN_rev	NoACTIN_rev	AACAACTCTCCTTCACA		Reverse
